# Electrochemical‐Genetic Programming of Protein‐Based Magnetic Soft Robots for Active Drug Delivery

**DOI:** 10.1002/advs.202503404

**Published:** 2025-04-29

**Authors:** Hang Zhao, Bo Yu, Dingyi Yu, Ting Ji, Kexin Nie, Jingyi Tian, Xinchen Shen, Kaiyue Zhang, Junhan Ou, Xinyi Yang, Dongfang Xiao, Qi Zhou, Wenwen Huang

**Affiliations:** ^1^ Centre for Regeneration and Cell Therapy The Zhejiang University‐University of Edinburgh Institute Zhejiang University School of Medicine Zhejiang University Hangzhou 310058 China; ^2^ Deanery of Biomedical Sciences Edinburgh Medical School College of Medicine and Veterinary Medicine The University of Edinburgh Edinburgh EH8 9XD UK; ^3^ Department of Orthopedics of the Second Affiliated Hospital Zhejiang University School of Medicine Zhejiang University Hangzhou 310058 China; ^4^ Dr. Li Dak Sum & Yip Yio Chin Center for Stem Cells and Regenerative Medicine Zhejiang University School of Medicine Zhejiang University Hangzhou 310058 China; ^5^ State Key Laboratory of Biobased Transportation Fuel Technology Zhejiang University Hangzhou 310027 China; ^6^ Biomedical and Health Translational Research Centre of Zhejiang Province Zhejiang University Hangzhou 310003 China

**Keywords:** de novo protein design, electrochemical engineering, in situ crystallization, stimuli‐responsive hydrogels, superparamagnetic soft robots

## Abstract

Magnetic soft robots have the potential to revolutionize the field of drug delivery owing to their capability to execute tasks in hard‐to‐reach regions of living organisms. Advancing their functionality to perform active drug delivery and related tasks necessitates the innovation of smart substrate materials that satisfy both mechanical and biocompatibility requirements while offering stimuli‐responsive properties. Optimization of the interaction between the substrate and magnetic components is also critical as it ensures robust actuation of the robot in complex physiological environments. To address these issues, a facile strategy is presented that synergistically combines genetic programming and electrochemical engineering to achieve on‐demand drug release with protein‐magnetite soft robots. As the substrate of the robot, genetically engineered silk‐elastin‐like protein (SELP) is encoded with thermo‐responsive motifs, serving as the dynamic unit to respond to temperature changes. Ultrafine magnetite (Fe_3_O_4_) nanocrystals are electrochemically nucleated in situ and grown on Fe‐protein coordination sites within the SELP hydrogel network, endowing reinforced mechanical strength, superparamagnetic property, and photothermal conversion capability. These soft robots can navigate confined spaces, target specific sites, and release drug payloads ex vivo in an intestinal model. Taken together, the proposed strategy offers an innovative approach to tailoring protein‐based soft robots toward precision drug delivery systems.

## Graphical abstract

This manuscript presents a protein‐based soft robot for active drug delivery. Stimuli‐responsive protein is rationally designed and processed into the substrate of robots. Superparamagnetic Fe_3_O_4_ nanoparticles are patterned on the substrate using an electrochemical method, enabling the robot to respond to external physical fields. The fabricated soft robots can navigate confined spaces, target specific sites, and release drug payloads on demand.

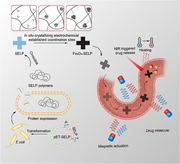

## 1 Introduction

Over 30 percent of drug candidates fail in clinical trials due to the fact that most drugs are delivered systemically which results in uncontrollable toxicity due to off‐target effects.^[^ ^1^ ^]^ Developing precise drug delivery strategies will facilitate the reconsideration of drugs that have been disqualified due to toxicity and help convert these promising therapeutics into successful therapies. Magnetic drug delivery soft robots hold great promise in clinical translations as they can deliver therapeutic and diagnostic agents into localized disease sites to minimize systemic toxicity.^[^ ^2^ ^]^ In general, current magnetic drug delivery soft robots release loaded drug molecules on‐site by passive diffusion or matrix degradation, showing limited control of the drug release profile. Progress toward smartness and functionality calls for the innovation of soft robot substrates, which can be responsive to physiological or external stimuli for on‐demand drug delivery. In addition, the clinical translations of such drug delivery soft robots largely depend on their biocompatibility and biodegradability.^[^ ^3^ ^]^ However, current widely adopted stimuli‐responsive substrates like poly(N‐isopropylacrylamide) (PNIPAM) cannot meet this demand, as their poor biodegradability and potential biotoxicity restrict their in vivo applications.^[^ ^4^ ^]^ Therefore, there is an unmet need to develop stimuli‐responsive substrates with intrinsic biocompatibility and biodegradability to realize the controlled on‐site on‐demand release of drug payloads.

Organisms have evolved a myriad of proteins with diverse functions to adapt to environmental changes, providing valuable inspiration for the design of new functional protein materials. Notably, many high‐performance proteins feature specific repetitive sequences.^[^ ^5^, ^6^ ^]^ For example, the composition of *Glycera* jaw proteins is enriched in glycine and histidine residues, with the sequence consisting of GGH/GGHG, GGHH, or HHG/GHH repeats. These repetitive sequences impart proteins with excellent biophysical and mechanical characteristics, such as remarkable toughness and wear resistance.^[^ ^7^ ^]^ The rapid development of synthetic biology has transformed the biomaterials manufacturing paradigm, enabling the *de novo* design of functional biomaterials with biocompatibility and biodegradability. Significant progress has been made in producing recombinant proteins with specific physicochemical properties through the rational design of amino acid sequences, according to the existing or newly predicted sequence‐function relationships.^[^ ^8^, ^9^, ^10^, ^11^ ^]^ These newly designed protein polymers can exhibit similar or even superior physicochemical properties when compared to their natural counterparts.^[^ ^12^ ^]^ Therefore, synthetic biology provides a design and production platform to obtain protein‐based polymers with desired stimuli‐responsive properties via rationally designing amino acid sequences.

Apart from stimuli‐responsive substrates, paramagnetic or ferromagnetic components can impart sensitivity and precision to external magnetic manipulation in soft robots. Among various magnetic materials, iron oxides like Fe_3_O_4_ are regarded as the most promising candidates for biomedical soft robots because of their simultaneous saturation magnetization, size‐dependent magnetic field responsiveness, and relatively low cytotoxicity.^[^ ^13^ ^]^ Usually, iron‐based oxides are processed into nanoparticles with a diameter of ca. 10 nm, as these particles exhibit superparamagnetic properties, which are crucial for the sensitive magnetic field responsiveness required in drug delivery applications.^[^ ^14^, ^15^, ^16^ ^]^ Moreover, the incorporation of these magnetic species into bulk materials is commonly achieved through physical absorption or entrapment of magnetic nanoparticles within the hydrogel matrix, which is prone to leaking due to weak bonding.^[^ ^17^ ^]^ Covalent bonding can improve the immobilization and control of magnetic components. However, such methods often rely on complex and time‐consuming ligand modification, with additional processes involving surfactants or other reactants.^[^ ^18^, ^19^ ^]^ Hence, developing convenient strategies to modulate the interaction between magnetic species and the polymer matrix is desired.

Electrofabrication has emerged as a promising additive manufacturing approach for processing hydrogel matrices. The application of electrical signals can direct the assembly and migration of polymer chains and guide the diffusion of ions created by electrochemically active electrodes.^[^ ^20^, ^21^, ^22^ ^]^ The electric field‐driven metal ions are capable of coordinating with the polymer to create localized crosslinking, thereby mechanically reinforcing hydrogels.^[^ ^20^, ^23^ ^]^ According to classical nucleation theory, these metal ion‐enriched coordination sites can serve as nuclei, providing attachment or absorption sites for subsequent crystal growth.^[^ ^24^ ^]^ Previous reports have demonstrated the feasibility of the direct growth of metal oxides on these coordination sites, indicating the potential for incorporating functionalized metal oxides into stimuli‐responsive hydrogels through in situ crystallization on the coordination sites.^[^ ^25^ ^]^ Furthermore, the type and size of synthesized metal oxide nanoparticles can be tailored by modulating different metal electrodes and the growth medium.^[^ ^26^ ^]^ Therefore, in situ crystallization of electrochemically established coordination sites represents a practical strategy for fabricating soft robots with integrated magnetic components.

**Scheme 1:**
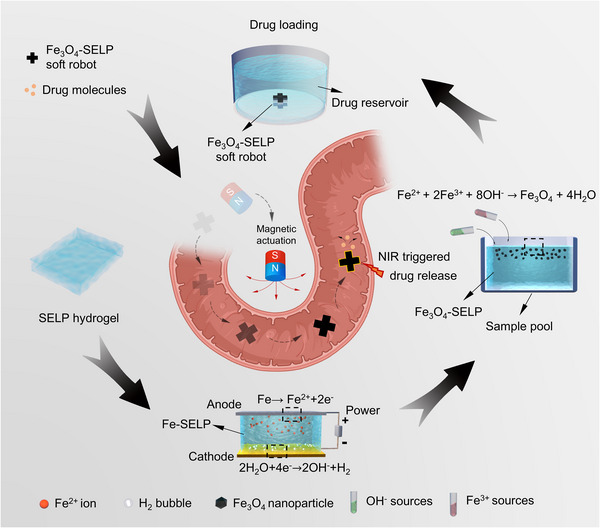
Schematic illustration of the design and application of Fe_3_O_4_‐SELP soft robots. Bilayer cross‐shaped Fe_3_O_4_‐SELP superparamagnetic soft robots were fabricated through in situ crystallization on electrochemically established coordination sites with drug loading/release capacity for active delivery. (Part of  Scheme 1 was created with BioRender.com).

Herein, we report the design of a protein‐based superparamagnetic soft robot with intrinsic biocompatibility, active targeting, and controllable drug release properties toward precision drug delivery (**Scheme** **1**). Genetically engineered silk‐elastin‐like proteins (SELPs) are designed and produced via synthetic biology technology, serving as the stimuli‐responsive hydrogel substrate. The elastin domain (GVGVP) in SELPs imparts elastic and thermo‐responsive properties, while the silk domain (GAGAGS) supports both drug delivery and suitable mechanical properties for robotics by modulating the degree of crystallinity. The Fe_3_O_4_ nanoparticles are selected as the power transmission media and are grown within the SELP network through in situ crystallizing electrochemical established coordination sites. By carefully regulating the growth conditions, Fe_3_O_4_ nanocrystals with uniform size (ca. 8 nm diameter) are integrated into the hydrogel network of the SELP substrate and thus enhance the mechanical properties through metal ion‐protein coordination. Furthermore, these ultrafine Fe_3_O_4_ nanocrystals exhibit superparamagnetic properties, endowing drug delivery soft robots with sensitive magnetic responsive properties. Additionally, due to the photothermal conversion ability of Fe_3_O_4_ nanocrystals, near‐infrared (NIR) irradiation can induce the contraction behavior of the SELP substrate to trigger drug release. The feasibility of using these robots for active targeting and on‐demand drug release is validated ex vivo in porcine intestine models. The biocompatibility of these drug delivery soft robots is also evaluated both in vitro and in vivo. This work presents an effective design of biocompatible magnetic soft robots for on‐demand delivery of therapeutics in otherwise inaccessible lesion sites, thereby addressing challenges and unmet needs in healthcare.

## 2 Results

### 2.1 Design Principles of Recombinant Stimuli‐Responsive Protein Hydrogel

**Figure 1:**
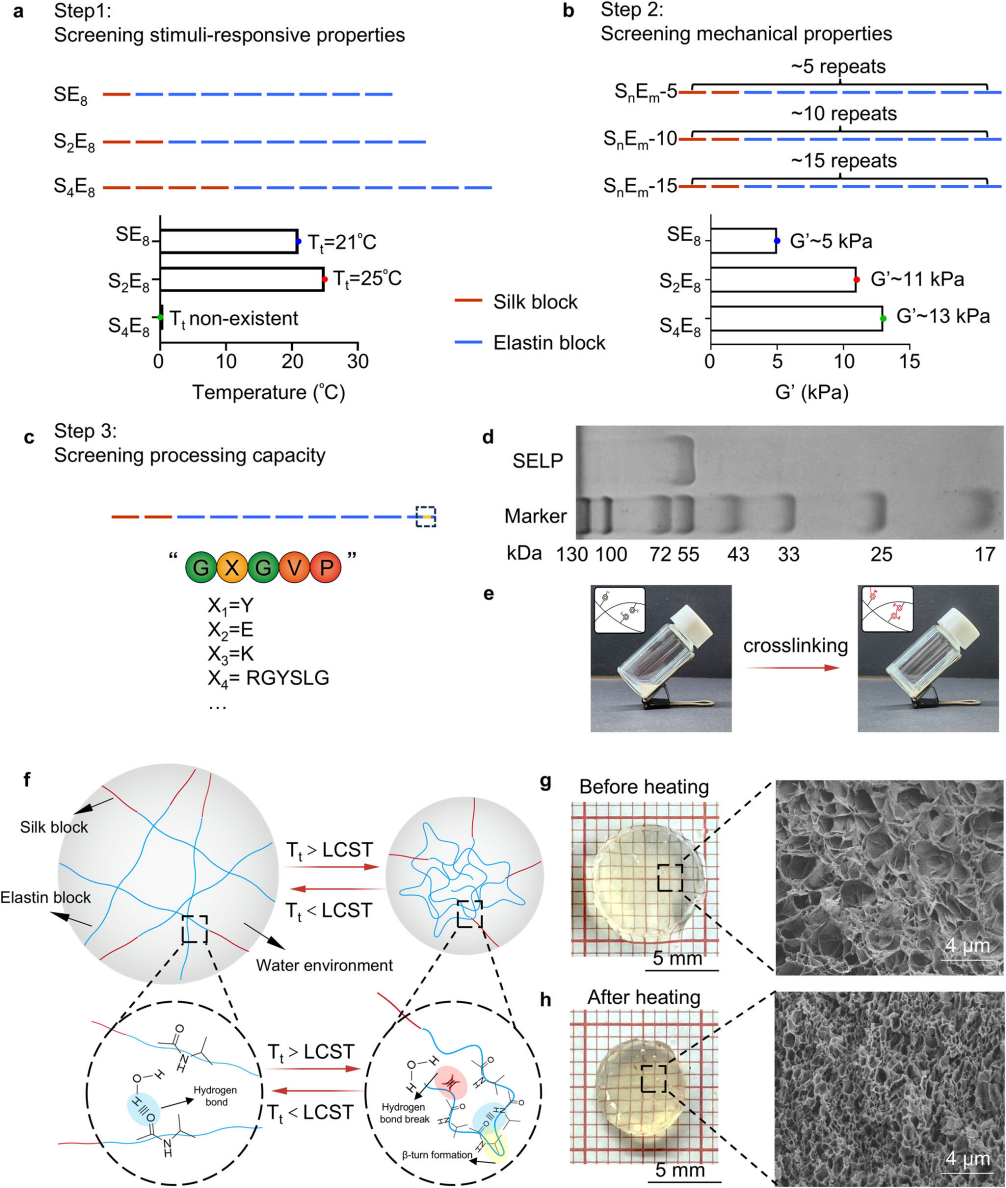
Molecular design, preparation, and characterization of stimuli‐responsive protein hydrogels. a–c) Design principles of the stimuli‐responsive protein polymers. d) SDS‐PAGE of purified recombinant proteins SELP using 10% SDS‐PAGE with Coomassie staining. e) Digital photograph of SELP before and after the di‐tyrosine crosslinking reaction. f) Schematic illustration of phase transition associated with lower critical solution temperature of SELP molecules. Digital photograph of SELP hydrogels and the associated SEM image g) before (4 °C) heat treatment and h) after (50 °C) heat treatment for 15 min.

Silk and elastin characteristic sequences were employed to construct stimuli‐responsive protein polymers to provide tunable mechanical and dynamic properties, respectively. SELP sequences were designated as S_n_E_mX_, where n, m, and X represented the silk block number, elastin block number, and characteristic amino acid in the elastin blocks, respectively. A series of criteria were utilized to optimize the gene sequences in the established library.^[^ ^27^, ^28^ ^]^ First, stimuli‐responsive properties were prioritized due to the fundamental requirement for controllable drug release from soft robots (**Figure** **1**a). Various silk‐to‐elastin ratios were screened and the results showed that sequences with a silk‐to‐elastin ratio below 1/2 retained the desired stimuli responsive properties. (Figure S1, Supporting Information). Next, the mechanical properties were evaluated as the second consideration. Herein, two strategies were employed to modify the mechanical performance of the protein sequences: altering the polymer chain length and adjusting the silk‐to‐elastin ratio (Figure 1b). Finally, the amino acid type situated at position “X” of the elastin domains was modulated to enhance the processing capacity of the hydrogel substrates (Figure 1c). Based on the above mentioned considerations and our previous studies,^[^ ^29^, ^30^ ^]^ S_2_E_8R_‐10 ([S_2_E_8R_, block copolymer design with silk (S) domains (GAGAGS)_2_ and elastin (E) domains (GVGVP)_4_ (RGYSLG) (GVGVP)_3_]‐10mer) were selected as the substrate due to its stimuli‐responsive properties, moderate mechanical properties, as well as rapid enzyme‐mediated gelation process. The recombinant proteins were expressed in *E.coli* and purified using the inverse transition cycling (ITC) method as previously reported.^[^ ^31^ ^]^ The molecular weight of purified SELP was measured by SDS‐PAGE (Figure 1d). The single band at ca. 50 kDa suggested that the ITC‐purified SELPs had high purity and the correct molecular weight.

SELP exhibits phase transition in an aqueous environment upon heating above the lower critical solution temperature (Figure S2, Supporting Information).^[^ ^29^, ^32^ ^]^ In order to convert this protein conformational transition into reversible macroscopic changes, di‐tyrosine crosslinking was employed to fabricate SELP hydrogels (Figure 1e). The fluorescence excitation‐emission spectra were collected to evaluate the formation of dityrosine bonds (Figure S3, Supporting Information), suggesting the transition from tyrosine to dityrosine bonds during the gelation process.^[^ ^31^ ^]^ Schematic illustrations and optical pictures shown in Figure 1f–h exhibited the states of the hydrogel before and after the phase transition. In the initial state, SELP is water‐soluble due to the hydrogen bonding interaction between the polymer chains and water molecules. Reflected on the macroscopic level, prepared hydrogels were optically clear (Figure 1g). Upon immersion in 50 °C aqueous solutions for 15 min, the SELP molecules undergo coacervation and induced the formation of β‐turns,^[^ ^33^, ^34^ ^]^ resulting in the structural transition to an aggregated state. Consequently, hydrogels became opaque and shrank in size (Figure 1h). Scanning electron microscopy (SEM) was employed to further characterize the phase transition behavior of SELP hydrogels. As shown in Figure 1g, lyophilized hydrogels possessed porous structures with an average pore size of ca. 4.3 µm before the phase transition. After heating, a smaller pore size was observed with an average pore diameter of ca. 0.5 µm (Figure 1h). The narrowed pore size distribution of the hydrogels was consistent with the loss of volume described above. In addition, as‐prepared stimuli‐responsive SELP hydrogels showed reversible contraction‐recovery performance over 20 cycles, indicating durable integrity and stable actuation (Figure S4, Supporting Information). Based on these results, we concluded that SELP hydrogels with thermal stimuli‐responsive properties were successfully designed and prepared, which could be used as the substrate for fabricating smart drug delivery soft robots.

### 2.2 Construction of Magnetic Soft Robots via Electrochemical Method

**Figure 2:**
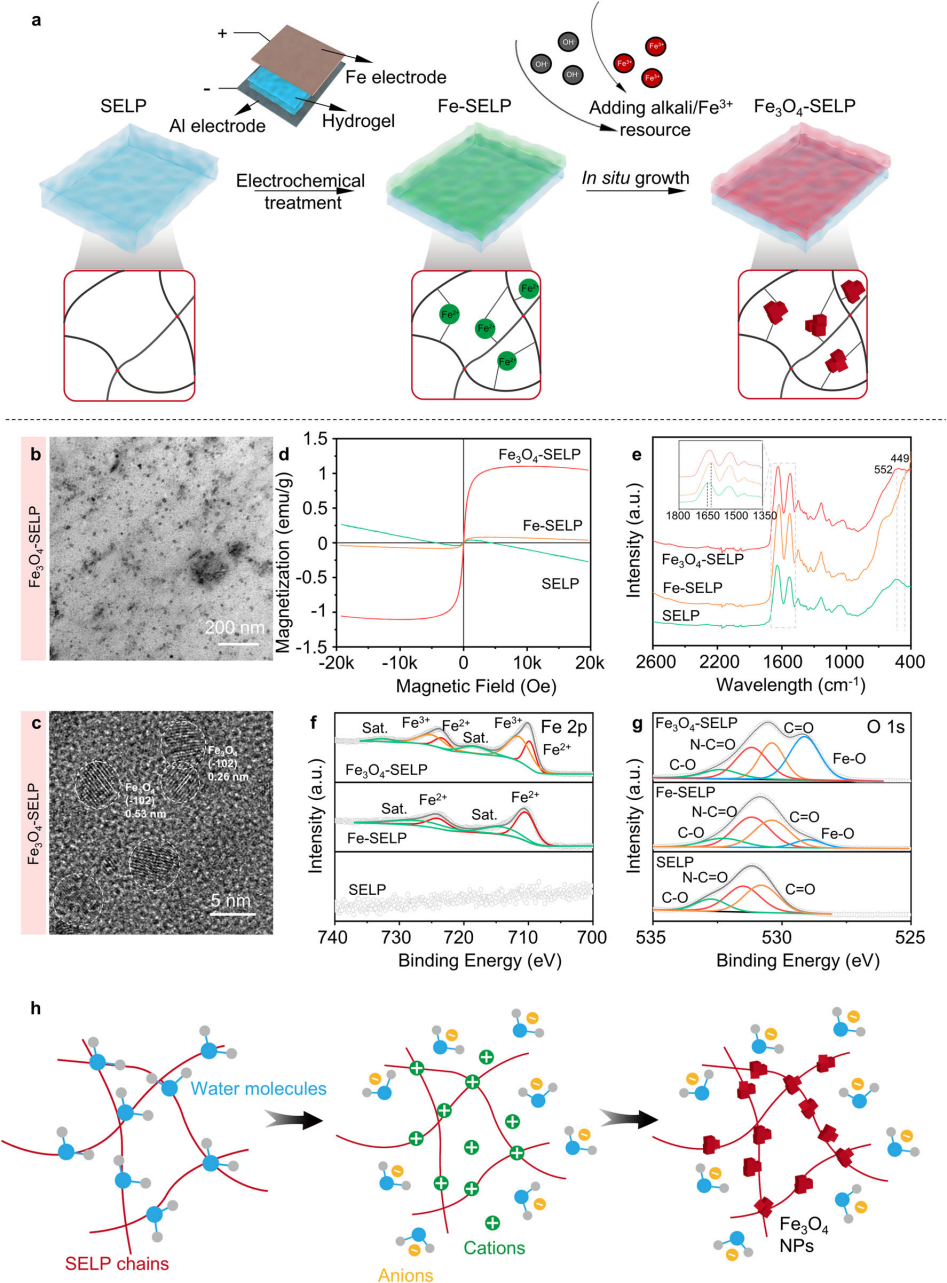
In situ electrochemical construction of ultrafine Fe_3_O_4_ nanoparticles on the SELP substrate. a) Schematic illustration of the in situ crystallization of Fe_3_O_4_ nanoparticles on the SELP substrate. b) TEM image of Fe_3_O_4_ nanoparticles dispersed in SELP hydrogel. c) High‐resolution TEM image of Fe_3_O_4_ nanoparticles. d) Magnetization curves of SELP, Fe‐SELP, and Fe_3_O_4_‐SELP at room temperature. e) FTIR spectra of SELP, Fe‐SELP and Fe_3_O_4_‐SELP. The insert image shows the magnified FTIR spectra of amide I and amide II bands. f) High‐resolution XPS spectra of the Fe 2p core levels in SELP, Fe‐SELP, and Fe_3_O_4_‐SELP. g) High‐resolution XPS spectra of the O 1s core levels in SELP, Fe‐SELP, and Fe_3_O_4_‐SELP. h) Mechanism illustration of in situ crystallization with electrochemically established coordination sites strategy.

Superparamagnetic iron oxide nanoparticles (SPIONs) were selected as the magnetic media of power transmission for soft robots and were grown in situ on the SELP substrates via in situ crystallization from electrochemically established coordination sites (Scheme 1 and **Figure** **2**a). Specifically, SELP hydrogels were sandwiched between an iron anode and an aluminum counter electrode. An oxidative bias was applied to the iron anode, releasing Fe^2+^ ions at the anode/hydrogel interface. These Fe^2+^ ions, driven by the electrical field, diffused into the hydrogel networks and coordinated with the protein chains, forming the Fe^2+^‐coordinated SELP hydrogels (Fe‐SELP). Acquired Fe^2+^‐protein coordination sites further served as the nucleation sites, facilitating the growth of the magnetic nanoparticles. After replenishing Fe^3+^ and alkali resources, SPIONs were generated in situ at the electrochemically treated areas, yielding Fe_3_O_4_‐SELP hydrogels. The chemical reaction involved in this step is:

(1) $$Fe^{2+}+2Fe^{3+}+8OH^{-}\to Fe_{3}O_{4}+4H_{2}O$$

In order to optimize this electrochemical process, different oxidative bias was employed to treat SELP hydrogels (Figures S5 and S6, Supporting Information). Taking into account the release kinetics of Fe^2+^ ions as well as the structural integrity of the hydrogel, 5 V was selected as the working voltage for subsequent tests. The proposed electrochemical strategy offers exceptional flexibility in the patterning of the magnetic species on the SELP hydrogel surface. Benefiting from the flexibility and compatibility of this method, magnetic soft robots can be programmed to execute pre‐defined motions by independently adjusting the geometry of the Fe electrode and SELP substrates, allowing for precise control of each component (Figures S7 and S8, Supporting Information). For convenience, the prepared bilayer soft robot that consisted of a SELP hydrogel layer and a Fe_3_O_4_‐SELP hydrogel layer was abbreviated as Fe_3_O_4_‐SELP robot.

X‐ray diffraction (XRD) was conducted to characterize the sample composition. As shown in Figure S9 (Supporting Information), after in situ Fe_3_O_4_ growth, several new peaks appeared in the Fe_3_O_4_‐SELP hydrogel diffraction pattern, which could be indexed to Fe_3_O_4_ (JCPDS No.28‐0491). The intensity of these peaks was not high, probably because the as‐prepared Fe_3_O_4_ was wrapped by the protein networks.^[^ ^35^ ^]^ To directly observe the Fe species in the hydrogel matrix and to further demonstrate the successful preparation of Fe_3_O_4_, transmission electron microscopy (TEM) was performed. Images showed that nanoparticles were uniformly distributed on the hydrogel skeleton with nanosphere morphology (Figure 2b). Besides, high‐resolution TEM (HRTEM) analysis revealed the crystalline structure of these nanoparticles (Figure 2c). The lattice fringes with crystal plane spacing of 0.26 nm correspond to the crystalline plane (‐102) of Fe_3_O_4_.^[^ ^36^ ^]^ This observation was consistent with the XRD observations in Figure S9 (Supporting Information). In addition, the size distribution of nanoparticles was analyzed by counting at least 100 nanoparticles using Image J. The average diameter of Fe_3_O_4_ was ca. 8±2 nm (Figure S10, Supporting Information). The magnetic properties of Fe_3_O_4_ are associated with particle size.^[^ ^16^ ^]^ The narrow size distribution of Fe_3_O_4_ imparts the hydrogel with superparamagnetic features, which is important for Fe_3_O_4_‐SELP robots to realize hysteresis‐free actuation. To demonstrate this property, a vibrating sample magnetometry (VSM) was employed to examine the magnetic properties of different samples (Figure 2d). At room temperature, SELP hydrogels were diamagnetic and Fe‐SELP hydrogels exhibited paramagnetic behavior with a saturation magnetization of 0.08 emu/g. After in situ Fe_3_O_4_ growth, the magnetization curve of Fe_3_O_4_‐SELP hydrogels showed S‐shaped hysteresis loops with a saturation magnetization of 1.10 emu g^−1^, confirming the superparamagnetic property of Fe_3_O_4_‐SELP samples. The saturation magnetization of Fe_3_O_4_‐SELP is noted to be smaller than that of commercial SPIONs. The moderate drop in magnetization could be ascribed to the decreased dipolar interactions as a consequence of enlarged interparticle distance.^[^ ^37^, ^38^ ^]^ Previous reports demonstrated that magnetic drug delivery carriers with saturation magnetization around 1.2 emu/g were still capable of precise targeting and cargo delivery.^[^ ^39^, ^40^ ^]^ Hence, Fe_3_O_4_‐SELP robots can be potentially harnessed for similar biomedical applications, with possibility for enhanced magnetization or stronger magnetic field.

To reveal the interaction between metal ions and the hydrogel networks during the Fe_3_O_4_ preparation process, Fourier transform infrared spectroscopy (FTIR) was carried out. As shown in Figure 2e, there were two strong absorbance peaks at amide I band (ca. 1610–1700 cm^−1^) and amide II band (ca. 1500–1570 cm^−1^) in SELP spectra, assigned to C═O stretching vibrations and in‐plane N─H bending, respectively.^[^ ^41^, ^42^ ^]^ Upon treatment with electrodes, the characteristic peak of amide I shifted from 1645 to 1625 cm^−1^ in Fe‐SELP spectra, suggesting the formation of Fe‐carbonyl coordinated interactions.^[^ ^43^ ^]^ Additionally, a new peak appeared at 449 cm^−1^, which could be ascribed to the Fe─O stretching vibration of Fe^2+^ placed in tetrahedral sites.^[^ ^44^, ^45^ ^]^ This result was also in accordance with the X‐ray photoelectron spectroscopy (XPS) data of Fe‐SELP hydrogels to be discussed in the following section. After introducing ions and alkali to initiate crystal growth, the amide‐I peak in Fe_3_O_4_‐SELP spectra shifted back to 1635 cm^−1^, indicating the change of the coordinating environment after crystal growth. Furthermore, a new peak emerged at 552 cm^−1^, attributed to the Fe─O stretching vibrations of Fe^3+^ ions located at the tetrahedral sites.^[^ ^46^ ^]^ These phenomena suggested that Fe_3_O_4_ grew in situ on the coordination sites.

Next, the surface chemical states of the samples at different processing stages were analyzed by XPS. SELP was used for comparison. Figure 2f shows the high‐resolution XPS spectra of the Fe 2p peaks. No iron‐related peaks were observed in the SELP samples. In contrast, the peaks at 710.4 and 724 eV were detected in Fe‐SELP, which could be assigned to Fe^2+^ 2p_3/2_ and Fe^2+^ 2p_1/2_.^[^ ^47^ ^]^ After the in situ growth procedure, the Fe^2+^ 2p peaks of Fe_3_O_4_‐SELP were observed at 710.1 and 723.7 eV, with new peaks at 711.9 and 725.5 eV corresponding to Fe^3+^ 2p_3/2_ and Fe^3+^ 2p_1/2_.^[^ ^48^ ^]^ Meanwhile, a strong satellite peak situated at around 719 eV could be attributed to the typical character of Fe^3+^.^[^ ^48^ ^]^ This result confirmed the coexistence of Fe(II) and Fe(III) states in the Fe_3_O_4_‐SELP samples, which is in good agreement with previously reported Fe_3_O_4_ spectra.^[^ ^48^ ^]^ Figure 2g exhibited the high‐resolution XPS spectra of the O 1s peaks. The asymmetric O 1s signal in SELP was deconvoluted into three peaks located at 530.8, 531.5, and 532.7 eV, which were associated with C═O, N─C═O, and C─O, respectively.^[^ ^49^, ^50^, ^51^ ^]^ However, in the Fe‐SELP, the peaks corresponding to the C═O bonds, N─C═O bonds, and C─O bonds were shifted to 530.4, 531.1, and 532.3 eV, respectively. This phenomenon may be attributed to the π‐back donation effect, which resulted in the movement of the π electrons toward the amide O atoms, thereby increasing their binding energy.^[^ ^52^, ^53^ ^]^ This result also demonstrated the interaction between Fe^2+^ and amide O atoms. Compared with SELP, the shift in binding energy provided evidence of the interactions between Fe^2+^ and amide oxygen atoms. Furthermore, a new peak emerged at 529.0 eV, which could be ascribed to Fe─O bonds.^[^ ^54^ ^]^ After the in situ growth, a strong peak of the metal‐O bond was observed in 529.1 eV, indicating further oxidation of ferrous species. The peaks related to the C═O bonds, N─C═O bonds, and C─O bonds in Fe_3_O_4_‐SELP were fitted at 530.5, 531.2, and 532.4 eV, respectively. These results demonstrate the successful in situ fabrication of ultrafine Fe_3_O_4_ nanoparticles by crystallization of Fe‐carbonyl coordination sites. Taken together, the mechanism of in situ crystallization of electrochemically established coordination sites can be explained as follows. The added NaCl solution imparts ionic conductivity to the SELP hydrogels. When SELP hydrogels are subjected to an appropriate electrical potential (5V, in this work), two electrode reactions occur in the cathode and anode, respectively, as follows:

(2) $$2H_{2}O+4e^{-}\to 4OH^{-}+H_{2}$$

(3) $$\mathrm{Fe}\to Fe^{2+}+2e^{-}$$

The consumed water molecules lead to an increase in the salt ion concentration in the hydrogel systems. Concentrated salt anions (Cl^−^, in this work) polarize the water molecules, disrupting the hydrogen interaction between the SELP chain and its bound water molecules. Meanwhile, Fe^2+^ ions produced at the anode/hydrogel interface can bind to amide oxygen atoms of SELP.^[^ ^43^, ^55^ ^]^ Fe‐carbonyl coordination sites were uniformly distributed at the anode/hydrogel interface, which formed the heterogeneous nucleation sites. After replenishing sufficient Fe^3+^ ions and alkali sources, ultrafine Fe_3_O_4_ nanocrystals formed on the electrochemically established coordination sites (Figure 2h).

### 2.3 Mechanics and Deformability of Fe_3_O_4_‐SELP Robots

**Figure 3:**
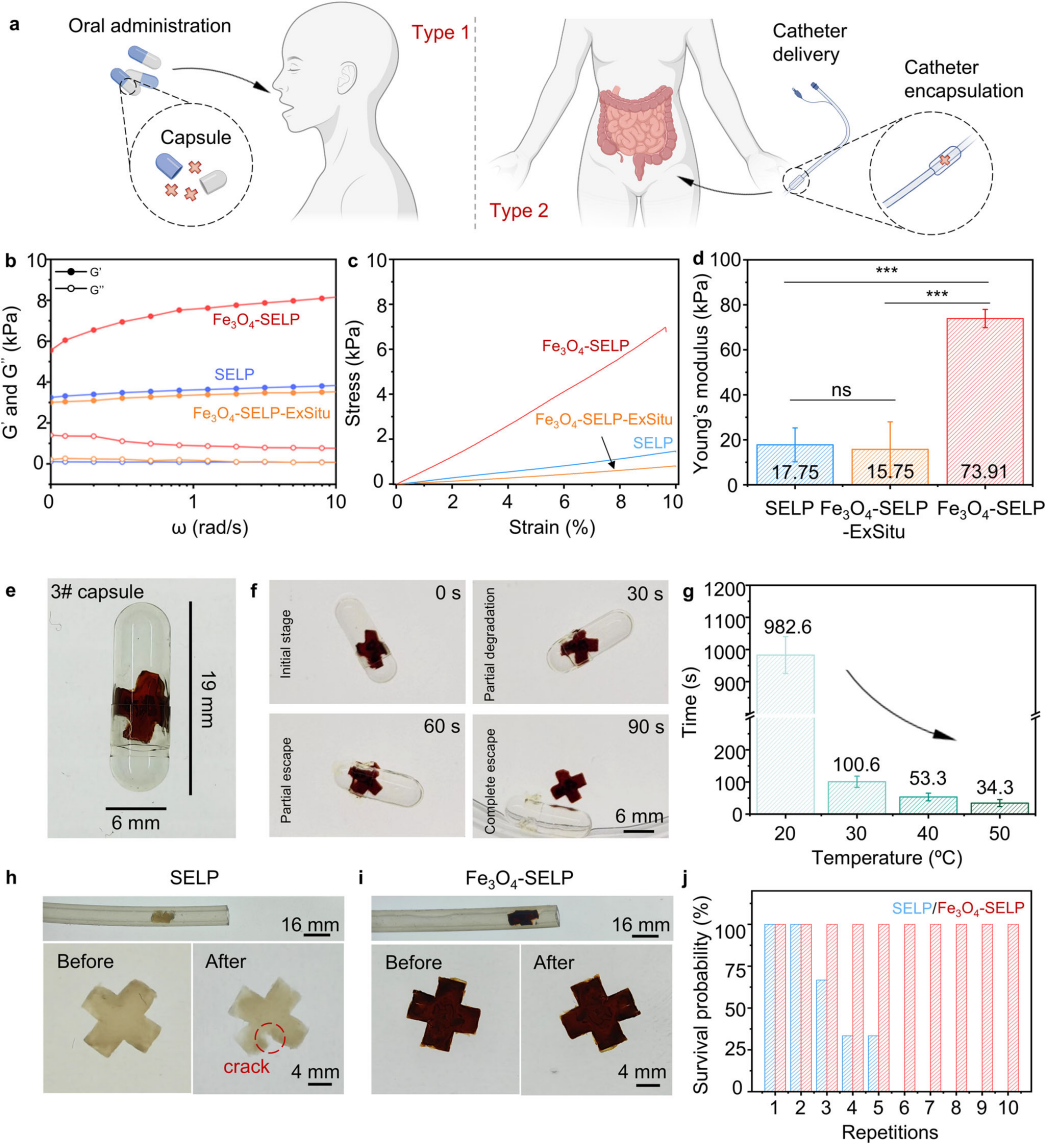
Navigation and transport in confined spaces enabled by enhanced mechanical properties. a) Schematic illustration of soft robot delivery strategies via oral administration or catheter delivery. b) Representative rheological frequency sweeps (storage and loss moduli) of SELP, Fe_3_O_4_‐SELP, and Fe_3_O_4_‐SELP‐ExSitu hydrogels. c) Compression stress‐strain curves of SELP, Fe_3_O_4_‐SELP, and Fe_3_O_4_‐SELP‐ExSitu hydrogels. d) The Young's modulus of SELP, Fe_3_O_4_‐SELP and Fe_3_O_4_‐SELP‐ExSitu hydrogels. e) A Fe_3_O_4_‐SELP robot inside a standard size “3” capsule (19.0 × 6.0 mm). f) Time sequence of Fe_3_O_4_‐SELP robot released from capsules. g) The relationship between temperature and robot release time. Representative images of h) SELP and i) Fe_3_O_4_‐SELP robots before and after release from catheter. j) The overall survival rate of SELP and Fe_3_O_4_‐SELP robots in repeated catheter encapsulation process. Parts of a) were created with BioRender.com. Data represents mean ± SD (n = 3). Significance was determined by one‐way ANOVA (***p<0.001).

Drug delivery soft robots should be mechanically robust to withstand complex physiological environments or excessive deformation to pass through confined spaces to enable the delivery of cargo to target sites in vivo (**Figure** **3**a). According to the in situ crosslink mineralization mechanism, the ultrafine Fe_3_O_4_ nanoparticles grown at the coordination sites should increase the mechanical properties of the hydrogels.^[^ ^25^ ^]^ To confirm whether in situ crystallized SPIONs can enhance the mechanical performance of the Fe_3_O_4_‐SELP hydrogels, Fe_3_O_4_‐SELP‐ExSitu hydrogels were prepared, in which Fe_3_O_4_ nanoparticles were fabricated in the hydrogels without applying electric field (details shown in “Experimental Section”). SELP hydrogels were also fabricated as control samples. Rheology measurements were carried out to evaluate the mechanical properties of all three types of samples. As shown in Figure 3b and Figure S11 (Supporting Information), the storage modulus G’ was higher than the loss modulus G’’ across the entire frequency range, indicating the solid‐like behavior of all hydrogels. The Fe_3_O_4_‐SELP hydrogel samples had the highest storage modulus among the three samples, followed by SELP and Fe_3_O_4_‐SELP‐ExSitu hydrogels in the entire frequency range. A similar trend was observed in the compression test. As shown in Figure 3c,d, the Fe_3_O_4_‐SELP hydrogels had the highest Young's modulus, which was 4.16 and 4.69 times higher than that of the SELP and Fe_3_O_4_‐SELP‐ExSitu hydrogels, respectively. We also compared the mechanical performance of Fe_3_O_4_‐SELP with conventional PNIPAM‐based systems. As shown in Table S1 (Supporting Information), the mechanical properties of Fe_3_O_4_‐SELP are superior to some reported PNIPAM‐based systems. The underlying mechanism for mechanical reinforcement can be attributed to the synergistic effect of electrochemically‐established coordination interactions and in situ Fe_3_O_4_ crystallization. Compared with pure SELP hydrogels, the established Fe‐carbonyl coordination sites enhance the crosslinking density of the SELP hydrogel network, whereas the in situ crystallization of Fe_3_O_4_ can contribute to the recruitment of elastically inactive SELP protein polymer chains to form additional networks to enhance the mechanical strength.^[^ ^25^, ^56^ ^]^

The improved mechanical properties of Fe_3_O_4_‐SELP hydrogel can endow soft robots with the ability to ingress and egress from confined spaces without causing damage, providing multiple strategies to deliver soft robots inside the body. Herein, two typical delivery approaches were selected to illustrate this point (Figure 3a). Oral administration is preferred to deliver soft robots due to the non‐invasive and pain‐free approach.^[^ ^57^ ^]^ Hence, capsules were chosen for the soft robots. As shown in Figure 3e, a bilayer cross‐shaped Fe_3_O_4_‐SELP robot with a length of 8 mm was successfully encapsulated into a 6 mm diameter capsule. We systematically assessed the relationship between the temperature and robot release time, and the encapsulated soft robot was released within 90 seconds when exposed to fluid with a viscosity of 1.2 cP at body temperature (Figure 3f,g; Movie S1, Supporting Information). Alternatively, a catheter was also used, as this is a desired medical apparatus to deliver fabricated soft robots to navigate through rugged and complex organs, reaching target sites without direct contact with the complex fluid environment of the human body.^[^ ^58^ ^]^ Herein, a silicon tube with an inner diameter of 4 mm was utilized to transport the soft robots. Cross‐shaped SELP robots and bilayer cross‐shaped Fe_3_O_4_‐SELP robots were loaded into the tube by vacuum and released with saline flow (Movies S2 and S3, Supporting Information). Figure 3h,i shows typical optical images of SELP robots and Fe_3_O_4_‐SELP robots before and after catheter delivery. No visible damage was observed on the Fe_3_O_4_‐SELP robots after this process, while the SELP robots showed defects such as cracks. Furthermore, the structure of Fe_3_O_4_‐SELP robots remained intact after at least 10 loading and release cycles (Figure S12, Supporting Information). We repeated this cycling encapsulation process with three individual samples in each group. The statistical consequence indicated the excellent structural stability of Fe_3_O_4_‐SELP robots (Figure 3j). These results demonstrated that Fe_3_O_4_‐SELP robots were compatible with typical delivery methods, indicating the advantages of the electrochemical method.

### 2.4 Multimodal Locomotion of Fe_3_O_4_‐SELP Robots

**Figure 4:**
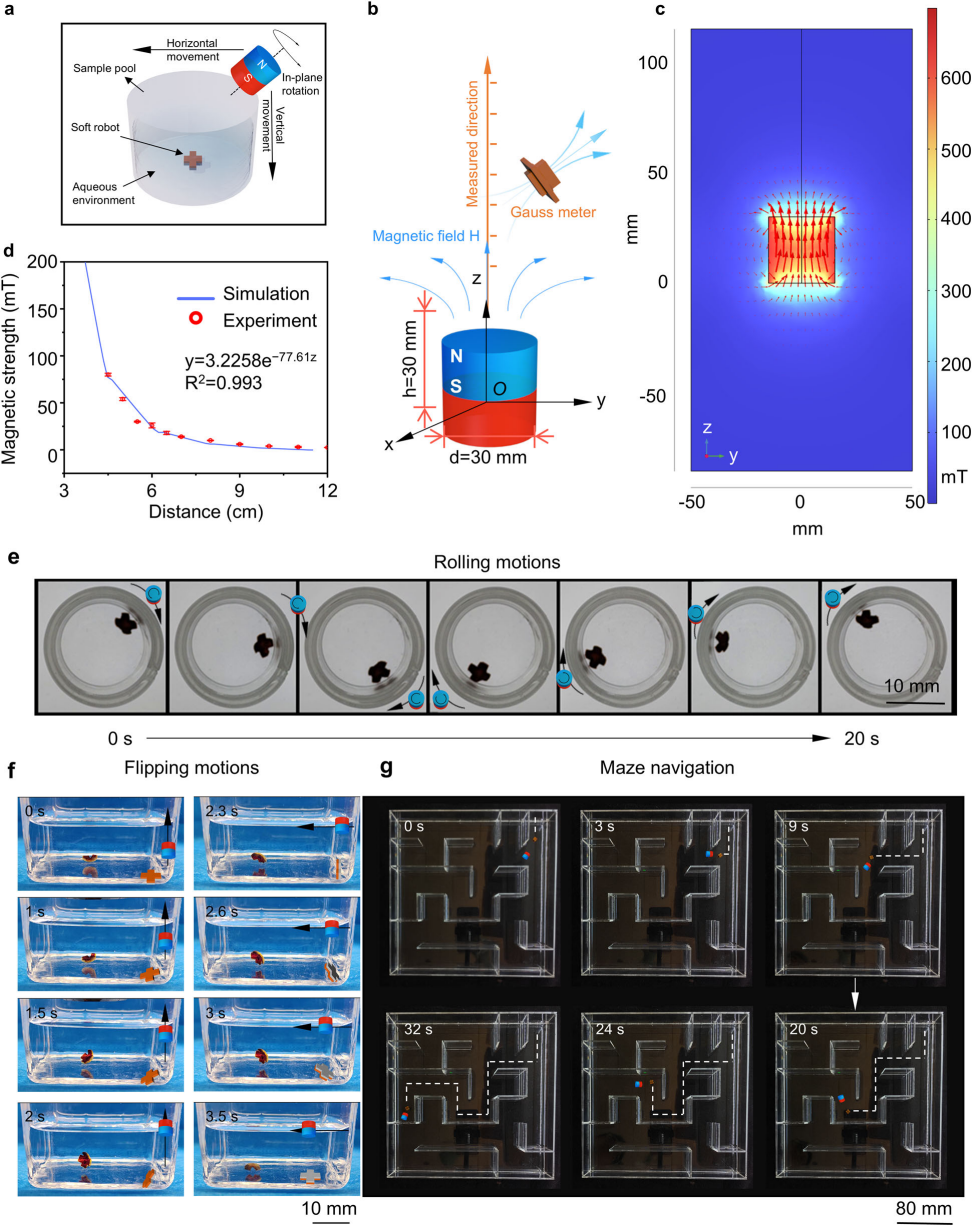
Magnetic control and multimodal locomotion of the magnetic soft robot. a) Schematic illustration of actuating the magnetic soft robot with a column permanent magnet. b) Schematic illustration of the column magnet with a residual magnetic flux density of B_r_ = 800 mT. c) Finite element analysis of the magnet field generated by the column magnet. d) The magnetic field strength H_z_ as a function of distance along the centerline of the magnet. e) Time sequence of the magnetically‐controlled rolling process. f) Time sequence of the magnetically‐controlled flipping process. g) Demonstration of real‐time maze navigation under magnetic guidance.

Targeting specific sites is the most fundamental and desired ability for drug delivery soft robots. These soft robots were expected to alter their motion and mode in order to fit into complex microenvironments in vivo and deliver drugs to desired sites. The presence of SPIONs on Fe_3_O_4_‐SELP robots allowed multiple motion modes by modulating the external magnetic field. In this study, a column magnet composed of NdFeB was adopted as an actuation resource (**Figure** **4**a). To better describe the magnet field distribution, a rectangular coordinate system was established with the center of the column magnet as the origin *O* and the axial, radial, and tangential directions as the z‐axis, x‐axis, and y‐axis, respectively (Figure 4b). Based on this coordinate system, a finite element analysis was conducted to visualize the magnetic field distribution (Figure 4c). Additionally, the magnetic field strength H_z_ was also calculated as a function of distance along the z‐axis, which was consistent with the values measured by a Gauss meter (Figure 4d). These results demonstrated that an external magnetic field gradient was produced by this actuation resource. We discussed the criterion for the Fe_3_O_4_‐SELP actuating, and the relevant analysis was expressed in Supporting Information. By manipulating the relative position of the soft robot with the NdFeB magnet, magnetic torques and forces were generated to drive the motion of the drug delivery robot.

Several typical motions, including rolling, flipping, and gliding, were selected and accomplished by tailoring external magnetic fields. Figure 4e and Movie S4 (Supporting Information) illustrated the rolling motions of Fe_3_O_4_‐SELP robots subject to a rotating magnet attraction. By rotating axially while moving the magnet clockwise, soft robots could realize rolling behavior along the sample pool. The angular displacement as a function of time was calculated and simulated using a linear fit, and the profile showed that the drug delivery soft robot achieved a steady rolling motion (Figure S13, Supporting Information). In another example, flipping behavior was accomplished by applying vertical and horizontal magnetic forces sequentially by the NdFeB magnet (Figure 4f; Movie S5, Supporting Information). These forces were modulated by regulating the position of the magnet along the y‐axis and x‐axis direction, contributing to the flipping process. Finally, a maze model was employed to assess gliding motion, as shown in Figure 4g and Movie S6 (Supporting Information). The Fe_3_O_4_‐SELP robot was guided through a maze by manipulating the NdFeB magnet. We randomized targeting sites in the maze at least 10 times and repeated the navigation process to evaluate the targeting efficiency. The results showed that Fe_3_O_4_‐SELP robot could reach the targeting location with 100% success rate (Figures S14 and S15, Supporting Information). We also assessed the velocity of the Fe_3_O_4_‐SELP robot in maze navigation (Figure S16, Supporting Information), the velocity of the Fe_3_O_4_‐SELP robot (7.48 cm s^−1^) was much higher than that of some physiological motions, for example, the peristaltic velocity of the intestine (0.5–2 cm s^−1^).^[^ ^59^ ^]^ In conclusion, the above‐mentioned results demonstrated that the soft robots propelled by magnetic fields were able to achieve multiple motion modes and navigate within complex routes, indicating their adaptability to the physiological environment and potential for biomedical applications.

### 2.5 NIR‐Triggered Drug Release of the Fe_3_O_4_‐SELP Robot

**Figure 5:**
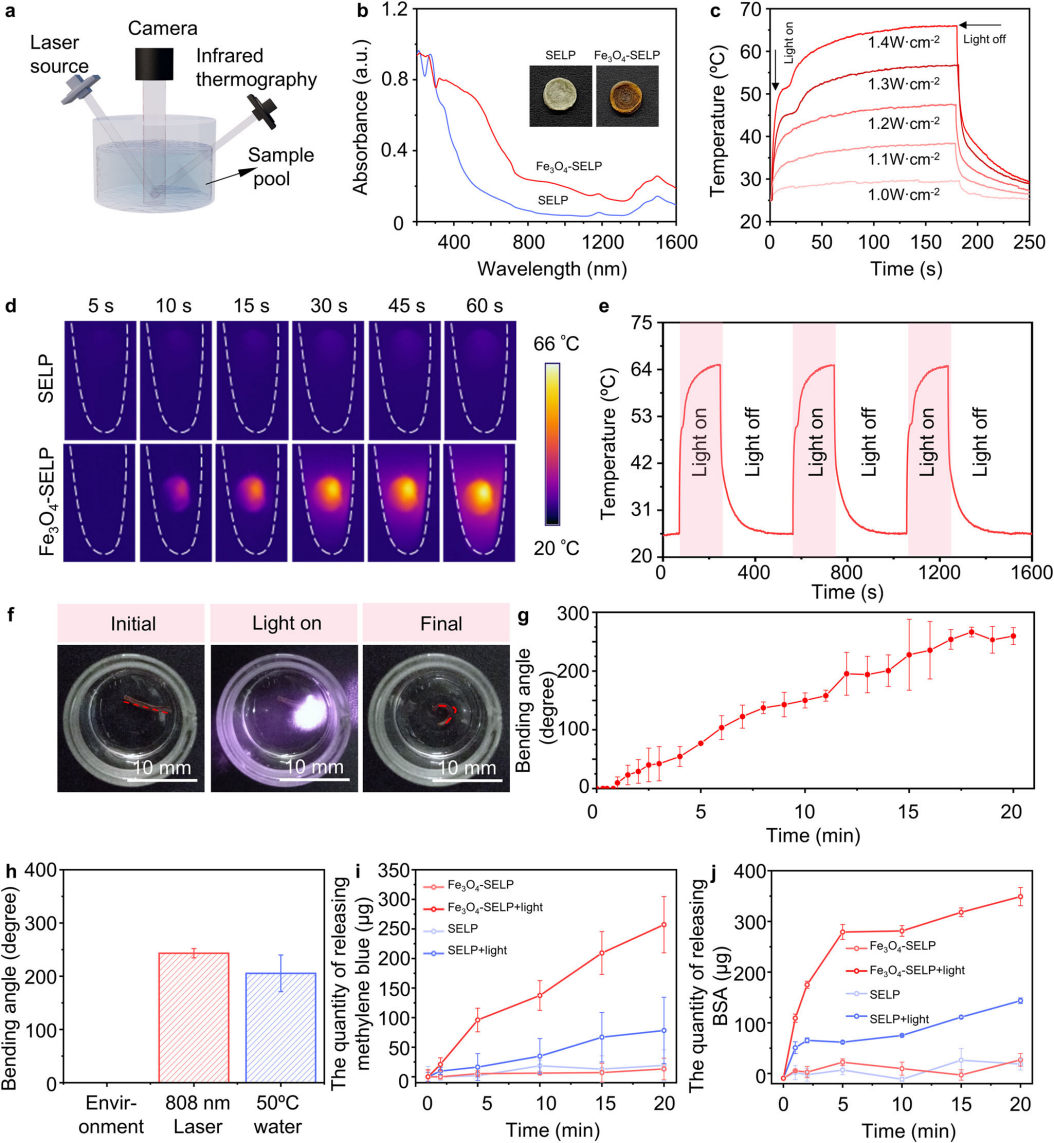
Photothermal properties and NIR‐triggered drug release of Fe_3_O_4_‐SELP robot. a) Schematic illustration of customised monitoring apparatus. b) UV‐vis‐NIR absorption spectra of SELP and Fe_3_O_4_‐SELP samples. c) Photothermal conversion of Fe_3_O_4_‐SELP hydrogels under 808 nm laser irradiation with different exposure intensities (1.0–1.4 W cm^−2^). d) Real‐time thermal images of SELP and Fe_3_O_4_‐SELP hydrogels under 808 nm laser irradiation (1.4 W cm^−2^). e) Photothermal stability of Fe_3_O_4_‐SELP hydrogels subject to three heating‐cooling cycles. f) The morphology of a bilayer stripe‐shaped Fe_3_O_4_‐SELP robot before (left), during (middle), and after (right) irradiation. g) The bending angle variation of stripe‐shaped Fe_3_O_4_‐SELP robots as a function of irradiation time under 808 nm laser irradiation (1.4 W cm^−2^). h) Maximum bending angle of Fe_3_O_4_‐SELP robot under different conditions. Release profiles of i) Methylene blue and j) BSA from SELP and Fe_3_O_4_‐SELP robots with or without 808 nm laser irradiation. Data represents mean ± SD (*n* = 3).

Apart from excellent mechanical and locomotion performance, precise control of drug release behavior is essential for drug delivery soft robots. The conventional method to induce actuation of SELP‐based hydrogels is to change the surrounding environmental temperature, which does not meet the demand for localized and controllable drug delivery in vivo.^[^ ^30^ ^]^ Beneficial to the unique properties of Fe_3_O_4_ nanoparticles, i.e., enhanced NIR light absorption and photothermal conversion ability,^[^ ^60^, ^61^ ^]^ Fe_3_O_4_ anchored SELP‐based soft robots enabled localized light‐driven drug release. To investigate the NIR‐triggered drug release behavior, a lab‐designed experimental setup was constructed, as illustrated in **Figures** **5**a and S17 (Supporting Information). This system was composed of a camera, infrared thermography, 808 nm laser, and a sample pool, enabling in situ monitoring of the multiple factors involved in this process, such as temperature variations and deformation of the soft robot. Next, the light absorption performance of SELP and Fe_3_O_4_‐SELP hydrogels was evaluated. Figure 5b shows the ultraviolet‐visible‐near‐infrared (UV‐vis‐NIR) absorption spectra of SELP and Fe_3_O_4_‐SELP hydrogels. The lyophilized SELP hydrogel exhibited only absorption in the UV irradiation range from 200 to 420 nm, while the lyophilized Fe_3_O_4_‐SELP hydrogel showed a strong absorption throughout the UV‐vis‐NIR wavelength range of 200–1600 nm. The enhanced light absorption of Fe_3_O_4_‐SELP hydrogels was ascribed to the anchored Fe_3_O_4_ nanoparticles. Then, the Fe_3_O_4_‐SELP hydrogels were exposed to 808 nm laser irradiation for a certain time to assess the photothermal conversion ability of Fe_3_O_4_, with the SELP hydrogels as the control group.^[^ ^62^ ^]^ As shown in Figure 5c, Fe_3_O_4_‐SELP hydrogels exhibited a time‐dependent change in temperature profile: the temperature increased rapidly from 25 to 50 °C within 12 s and reached equilibrium at around 64 °C after 180 s light irradiation. An immediate temperature drop was observed once the light source was turned off. Meanwhile, the temperature increment results of the Fe_3_O_4_‐SELP hydrogels irradiated with different powerscycles from 808 nm laser demonstrated that the ultimate equilibrium temperature of Fe_3_O_4_‐SELP hydrogels increased significantly with the increase of the power of light. Much smaller temperature changes were observed in the SELP hydrogel samples (Figure S18, Supporting Information), implying that the SELP has limited photothermal conversion capability. The representative images of two samples during the temperature‐increasing stage were recorded by infrared thermography and presented in Figure 5d. Additionally, Fe_3_O_4_‐SELP hydrogels exhibited excellent stability in terms of photothermal conversion, as evidenced by the fact that the maximum temperature remained unchanged after multiple “on‐off” cycles under light irradiation of 1.4 W cm^−2^ (Figure 5e). Using a previously reported method,^[^ ^63^ ^]^ the photothermal conversion efficiency of Fe_3_O_4_‐SELP at 808 nm was calculated to be 21.7%, showing the potential to control the localized drug delivery in vivo (Figure S19, Supporting Information).

An elevated temperature could drive the coacervation of SELP molecules, inducing the contraction of hydrogel networks.^[^ ^29^ ^]^ In order to better observe this light‐responsive conformational transition, A bilayer stripe‐shaped Fe_3_O_4_‐SELP robot was customized since the degree of the deformation of the striped soft robots could be visualized and assessed by measuring the bending angle. Figure 5f exhibits several typical images extracted from this process. In the initial stage, the soft robot showed a flat shape in the aqueous environment at room temperature. After irradiation with the 808 nm laser, the soft robot bent toward the SELP side as the protein molecules folded, while the Fe_3_O_4_‐anchored side retained the initial shape. This transformation was also evident by the change in pore size before and after laser irradiation (Figure S20, Supporting Information). Based on the asymmetric stress distribution, the bilayer stripe‐shaped Fe_3_O_4_‐SELP robot changed from flat to U‐shaped. Quantitative measurements of the change in bending angle with time were conducted, and the relevant plot is shown in Figure 5g and Figure S21 (Supporting Information). A trend of gradually increasing bending angle from 0 degrees to ≈256 degrees was seen in the bilayer stripe‐shaped Fe_3_O_4_‐SELP robot, indicating the sustained light‐driven changes for the soft robot. The maximum bending level of photothermal deformation was comparable to that in 50 °C aqueous environment (Figure 5h).

To investigate drug loading and release properties of the Fe_3_O_4_‐SELP robots, experiments were carried out with SELP soft robots as the control group. Methylene blue (MB) and bovine serum albumin (BSA) were selected as model small‐molecule and macromolecular drugs, respectively. The MB loading performance of the SELP and Fe_3_O_4_‐SELP robots was investigated by immersing the soft robots into MB solutions with different initial concentrations (0.0625, 0.125, 0.25, and 0.5 mg mL^−1^) overnight. The quality of MB loading was calculated by measuring the remaining concentration of MB in the solution using a microplate reader at 664 nm. As shown in Figure S22 (Supporting Information), at different initial concentrations (0.0625, 0.125, 0.25, and 0.5 mg mL^−1^), the loading of MB in the Fe_3_O_4_‐SELP robots was around 5.12, 6.56, 106.82, and 387.90 µg, respectively, which was comparable with that of the SELP soft robots (11.73, 20.97, 113.29, and 316.15 µg). This approach was also adopted to test the BSA loading performance of the soft robots. The loading amount of BSA in the Fe_3_O_4_‐SELP robot was ca. 225.39, 390.96, and 729.28 µg at the different initial concentrations (0.25, 0.5, and 1 mg mL^−1^, respectively), which was slightly lower than that of the SELP robots (284.21, 457.91, and 789.43 µg) (Figure S22, Supporting Information). Next, the NIR‐triggered drug release behavior of Fe_3_O_4_‐SELP robots was evaluated. As shown in Figure 5i and Figure S23 (Supporting Information), the efficiency of MB release from the SELP and Fe_3_O_4_‐SELP robots without NIR irradiation was negligible, with only 19.36 and 13.26 µg released in 20 min, respectively. Upon exposure to NIR irradiation, the Fe_3_O_4_‐SELP robot exhibited a significantly increased MB release rate, with 257.17 µg drug release over the same period. The enhanced rate of drug release was attributed to the photothermal conversion due to the anchored Fe_3_O_4_ nanoparticles. Interestingly, the SELP robot also showed enhanced drug release under NIR irradiation (78.34 µg in 20 min), and this phenomenon may be associated with the thermal effect of NIR light. In the case of BSA (Figure 5j), the release efficiency of SELP and Fe_3_O_4_‐SELP robots without NIR irradiation was also negligible (18.58 and 26.99 µg in 20 min, respectively). In contrast, a rapid release was observed under NIR irradiation in the Fe_3_O_4_‐SELP robot. The BSA release rate from the Fe_3_O_4_‐SELP robot was high within the first 5 min (279.11 µg of BSA released) and then exhibited a stable slow release (348.94 µg of BSA released in 20 min). A similar release profile with NIR irradiation was seen in the SELP group, with a total of 143.56 µg of BSA released in 20 min. Compared with SELP and Fe_3_O_4_‐SELP robots, there was negligible release of loaded drugs from silk hydrogels with or without NIR irradiation (Figure S24, Supporting Information). All of these experimental results demonstrated that Fe_3_O_4_ nanoparticles grown on the SELP networks endowed soft robots with an excellent photothermal conversion ability to enable NIR light as a switch for the control of drug release, which can be harnessed for the treatment of acute diseases where localized administration of high‐dose drug is desired.^[^ ^64^ ^]^

### 2.6 Ex Vivo Navigation and Drug Release of Fe_3_O_4_‐SELP Robot in Intestinal Model

**Figure 6:**
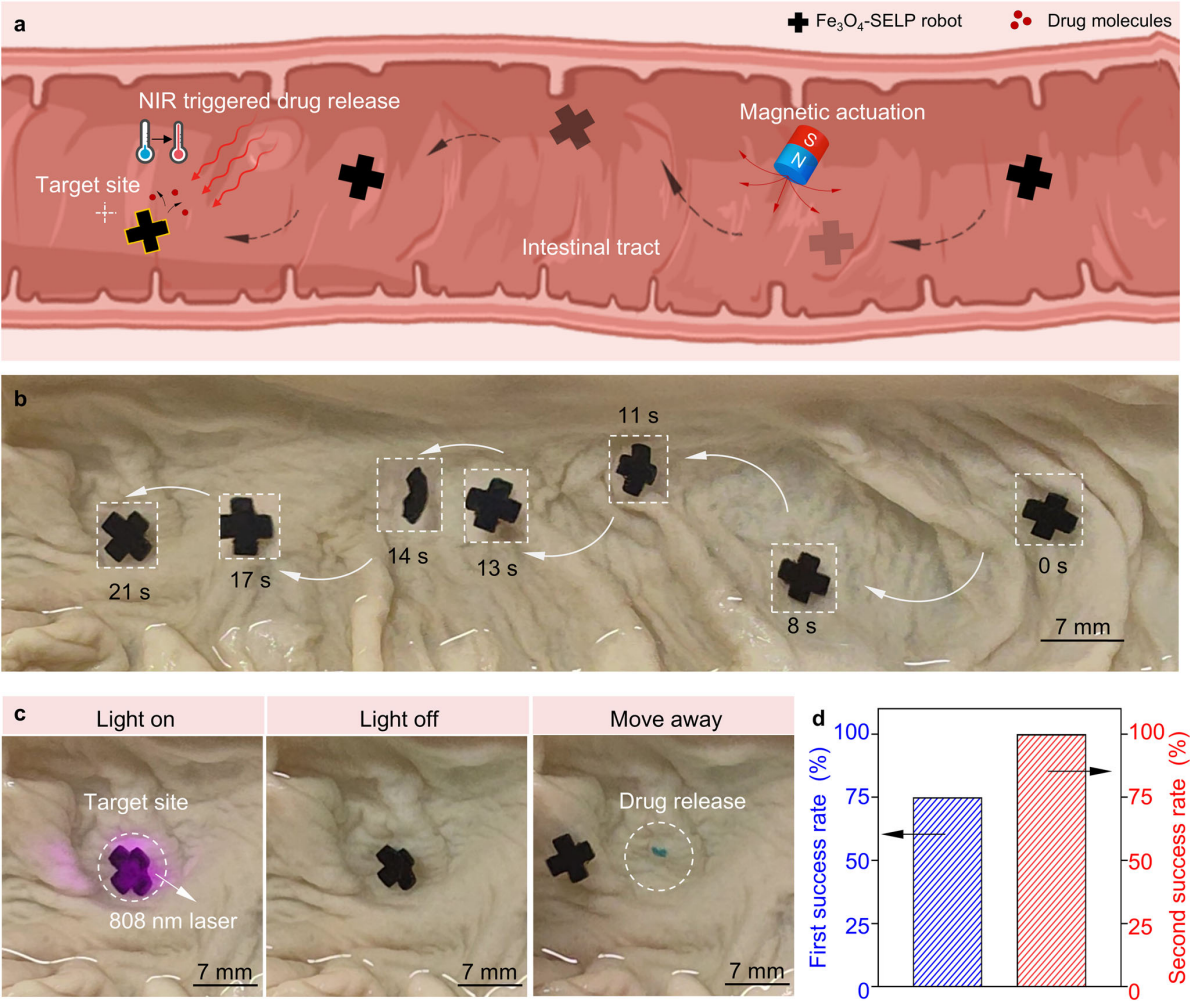
Ex vivo demonstration of magnetically‐controlled locomotion and NIR‐triggered drug release of Fe_3_O_4_‐SELP robot in a porcine intestinal model. a) Schematic illustration of the workflow for active drug delivery magnetic soft robot operating in the intestinal tract. b) Time‐series photographs of the targeted robot navigation. c) Representative photographs of the NIR‐triggered drug release process. Purple light: 808 nm laser spot; Blue dye: Methylene blue. d) The first success rate (smooth single attempt) and the second success rate (with local adaptation) of the Fe_3_O_4_‐SELP robot movement in the intestine.

To demonstrate the applicability of the drug delivery soft robot, the bilayer cross‐shaped Fe_3_O_4_‐SELP robot was controlled to target the specific site and release drugs in a porcine intestinal tract ex vivo. As shown in **Figure** **6**a, the ex vivo intestinal tract presented a physiological environment with different types of barriers, such as wrinkle areas, narrow channels, and mucus layers. PBS buffer was injected into the intestinal tract to mimic the intestinal fluid environment. Further, a targeted disease site was set to simulate a lesion site, and Methylene blue was selected as the model drug and loaded into the soft robots to visualize the drug release process. The magnetic field was generated by the NdFeB magnet at a vertical distance of ca. 4 cm, which was enough to externally control the Fe_3_O_4_‐SELP robot within the intestinal tract.^[^ ^65^, ^66^ ^]^ By controlling the NdFeB magnet, the soft robot was able to glide on the surface of the solution, flip through the wrinkles, and reach the targeted site (Figure 6b). When the bilayer cross‐shaped Fe_3_O_4_‐SELP robot successfully targeted the preset position (21 s), the 808 nm laser was used to induce the contraction of the SELP layer in the Fe_3_O_4_‐SELP robot, leading to the controlled release of the loaded drug as evidenced by the blue color (Figure 6c). During the whole process within the ex vivo intestinal tract, occasional stops in the targeting stage were observed owing to adhesion, which was addressed by changing the motion modes (Movie S7, Supporting Information). To better describe this phenomenon happened in targeting stage, we defined the first success rate and the second success rate as the probability of reaching the specified position at once and the probability of reaching the specified position after adjustment, respectively. The targeting sites were randomly set in the ex vivo intestinal model and the navigation process was repeated at least 15 times to assess the targeting efficiency quantitatively. Fe_3_O_4_‐SELP robot could reach the targeting location with 75% first success rate (Figure 6d). After adjustment, Fe_3_O_4_‐SELP robot was able to target to specific site with 100% success rate (Figure 6d). In addition, we also repeated the entire experiments containing targeting stage and drug release stage, and the Fe_3_O_4_‐SELP robot was able to consistently target the desired site and release the drug in a controllable fashion. These results demonstrated the control of drug delivery soft robots in the ex vivo intestinal model, indicating the potential for treating diseases in complex physiological environments like the intestinal tract.

### 2.7 Biocompatibility and Biodegradability

**Figure 7:**
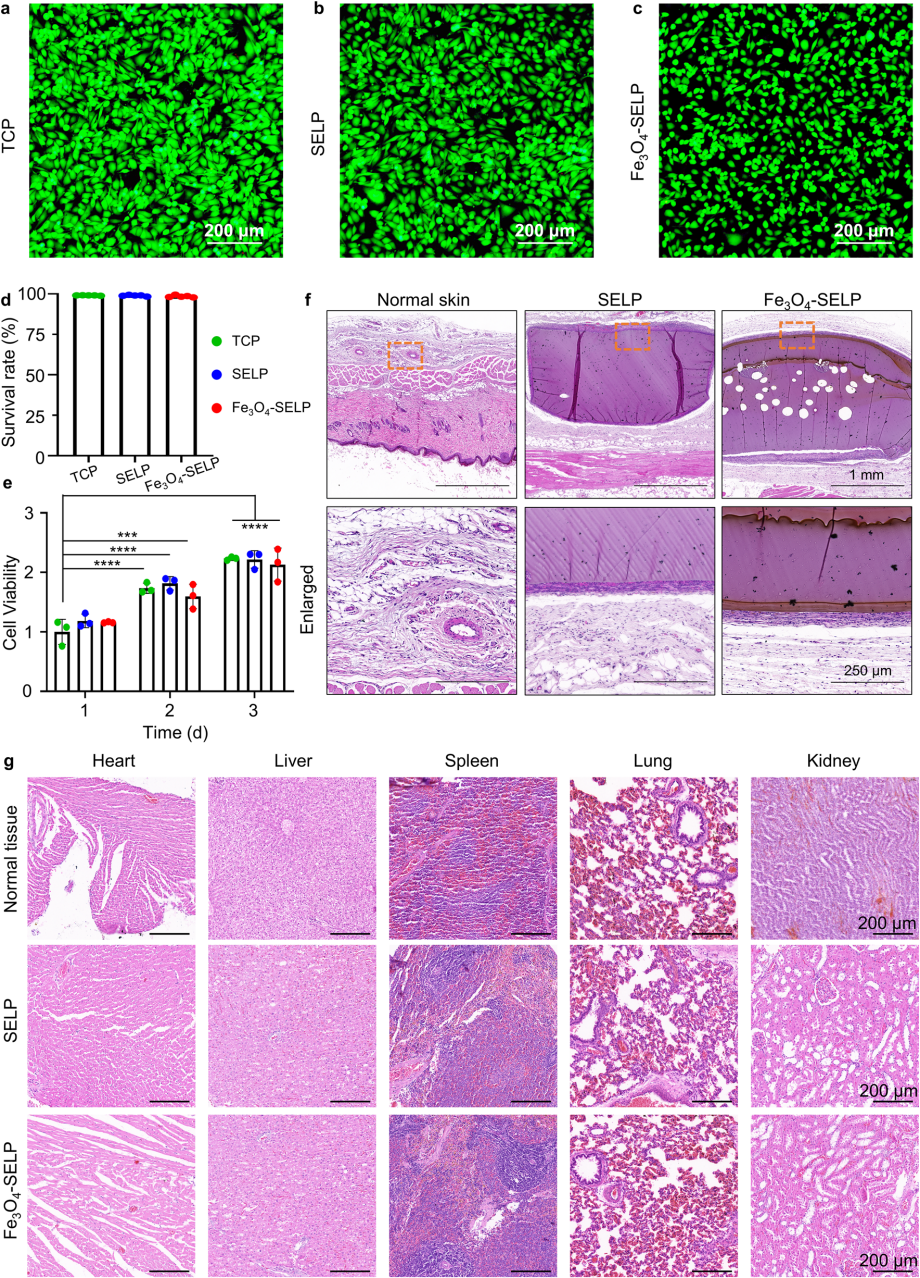
Biocompatibility and biodegradability of the Fe_3_O_4_‐SELP robot. Merged images of live (green) and dead (red) HUVECs in a) TCP, b) SELP, and c) Fe_3_O_4_‐SELP groups for 24h. d) The survival rate of HUVECs in TCP, SELP, and Fe_3_O_4_‐SELP groups. e) Cell viability of HUVECs after 1, 2, and 3 days. f) H&E staining of the normal tissue and tissues embedded with hydrogels after 4 weeks. g) H&E staining of the major organs (heart, liver, spleen, lung, and kidney) after 4 weeks. Data represents mean ± SD (*n* = 3). Significance was determined by one‐way ANOVA (^***^ *p*<0.001, ^****^ *p*<0.0001).

To evaluate the cytocompatibility of these drug delivery soft robots, the extract media of SELP and Fe_3_O_4_‐SELP hydrogels were collected. Human umbilical vein endothelial cells (HUVECs) were cultured in these extract media, and the tissue culture plate (TCP) extract was used as the control. After culturing the cells for 24 h, three groups mentioned above were stained with live/dead cell assay kits to evaluate cell viability. Lmedia ive cells were stained green (Calcein acetoxymethyl ester, Calcein‐AM), and dead cells were labeled red (propidium iodide, PI). **Figure** **7**a–c illustrates representative merged live/dead staining images of TCP groups, SELP groups as well as Fe_3_O_4_‐SELP groups, respectively. The majority of HUVECs were alive, and only a limited number of dead cells were noted. Individual images of stained HUVECs with different extract media were exhibited in Figure S25 (Supporting Information). The survival rate of HUVECs in different groups was calculated by ImageJ, and the statistical results were 99.07%, 98.96%, and 98.50% for the TCP, SELP, and Fe_3_O_4_‐SELP groups, respectively (Figure 7d). These high survival rates validated the cytocompatibility of the SELP and Fe_3_O_4_‐SELP hydrogels. In addition, Cell Counting Kit‐8 (CCK‐8) experiments were carried out to investigate cell proliferation. For this purpose, HUVECs were treated with the extraction solution collected from the TCP, SELP, and Fe_3_O_4_‐SELP samples for 1, 2, and 3 days, and the results are exhibited in Figure 7e. On the first day, the cell viability of the SELP group (1.18 fold) and Fe_3_O_4_‐SELP group (1.15 fold) was comparable to that of the TCP group. On the second and third days, it was observed that the viability of all three groups increased with culture time. Specifically, the cell viability of the Fe_3_O_4_‐SELP group was enhanced compared with the TCP group, i.e., day1: day1 (1.15 fold) < day2 (1.59 fold) < day3 (2.13 fold). These results showed that Fe_3_O_4_‐SELP hydrogel extract had no significant negative effect on the proliferation of HUVEC.

As protein‐based drug delivery soft robots, Fe_3_O_4_‐SELP robots can degrade into peptides or amino acids in response to proteolytic enzymes, and then be absorbed or metabolized in vivo.^[^ ^67^ ^]^ In order to evaluate the biodegradability of the Fe_3_O_4_‐SELP robots, trypsin was chosen as the enzyme to treat them, and the SELP robots were selected as the control group. As shown in Figures S26 and S27 (Supporting Information), both samples degraded in the presence of trypsin. SELP degraded completely within 8h. Compared with the SELP group, Fe_3_O_4_‐SELP robots showed a slower degradation, which fully degraded after 120h. This could be explained by the enhanced interaction between the SELP networks and SPIONs grown at the coordination sites.

Additionally, subcutaneous implantation of samples in a rat model was employed to assess the biocompatibility of hydrogels in vivo. Rats were surgically implanted with SELP and Fe_3_O_4_‐SELP hydrogels. After 4 weeks, the rats were sacrificed, and tissues in contact with the hydrogels were harvested. Hematoxylin and eosin (H&E) staining was performed and the results showed that there was no significant immune cell activity detected at the interface of the SELP and Fe_3_O_4_‐SELP hydrogels. Slight degradation occurred at the edge of the hydrogel, and significant cell proliferation was observed at the inter‐hydrogel space. This phenomenon was consistent with other reported protein‐based hydrogels (Figure 7f).^[^ ^68^ ^]^ Besides, H&E staining analysis of major organs showed that no obvious signs of histological differences and adverse inflammatory reactions were found in the major organs, confirming the excellent biosafety of SELP and Fe_3_O_4_‐SELP (Figure 7g). In conclusion, the above results demonstrated the ideal biocompatibility and biosafety of SELP and Fe_3_O_4_‐SELP, holding promise for further clinical translations.

## 3 Conclusion

In summary, we successfully constructed protein‐based hydrogel soft robots for active drug delivery. The stimuli‐responsive protein substrate was rationally designed and fabricated through synthetic biology technology, with recombinant proteins capable of responding to temperature changes being processed into hydrogels to serve as the dynamic units. To functionalize the soft robots with magnetic properties, a facile electrochemical method was developed to create metal ion‐protein coordination sites in the hydrogel networks for anchoring ultrafine metal oxide nanoparticles (in this study, Fe_3_O_4_ nanoparticles) via in situ crystallization. These anchored nanoparticles not only mechanically reinforce the hydrogel networks of the soft robot to allow versatile locomotion across confined spaces without damage, but also impart superparamagnetic property to the robot for remote control and targeted delivery.^[^ ^69^, ^70^ ^]^ Benefiting from the excellent photothermal conversion capacity of the ultrafine magnetite nanoparticles, the as‐prepared magnetic soft robots can respond to NIR irradiation to release loaded drug molecules in a controlled manner. Ex vivo experiments demonstrate the feasibility of targeted navigation and on‐demand drug release, thereby bearing potential for active drug delivery. In addition, in vitro and in vivo tests suggest excellent biocompatibility and biodegradability of these magnetic soft robots. Beyond the Fe_3_O_4_‐SELP robot, our work presents an effective design strategy for devising biocompatible and biodegradable magnetic soft robots towards active delivery of therapeutics aimed at unmet medical needs. For instance, in the treatment of acute diseases, such as acute seizures and acute pain, it is often required to administer high doses of drugs into the body rapidly. Protein‐based magnetic soft robots following our protocol could be rapidly guided towards the designated sites to release the drug molecules for alleviating acute symptoms. By optimizing the interaction between the drug molecules and hydrogel networks, such protein robots could enhance their payload retention ability for potential chronic disease management, e.g., diabetes and postoperative cancer care. It is also important to improve the efficiency of drug internalization since numerous drug molecules exert their functions by interacting with intracellular targets. Future work should focus on exploring the possibility of incorporating active targeting moieties, including tumor‐targeting peptides, cell penetration peptides, and antibodies, to further realize precision drug delivery at the cellular level.

## 4 Experimental Section

### Chemicals and Materials

Fe and Al plates were purchased from Xinji Metal Materials Co., Ltd. (China). Ampicillin, horseradish peroxidase (HRP), Luria‐Bertani (LB) culture medium, isopropyl‐β‐D‐thiogalacto‐side (IPTG), and imidazole were purchased from Sangon Biotech (Shanghai, China). Restriction endonucleases and T4 DNA ligase were obtained from New England Biolabs (Ipswich, MA). Chemically competent cells of *Escherichia coli* BL21(DE3) serving as protein expression hosts were obtained from WEIDI Biotech (Shanghai, China). Sodium hydroxide (NaOH), iron chloride, ethanol, hydrogen peroxide (H_2_O_2_), and sodium dodecyl sulfate (SDS) were purchased from Sinopharm Chemical Reagent Co., Ltd. Cell Counting Kit‐8 was purchased from Yeasen Biotechnology (Shanghai) Co., Ltd. Porcine intestinal tract was obtained from a local slaughterhouse.

### Recombinant Protein SELP Expression and Purification

The expression and purification of the SELPs were conducted based on the previous report.^[^ ^28^ ^]^ Briefly, the multimer gene SELP [(GAGAGS)_2_(GVGVP)_4_(RGYSLG)(GVGVP)_3_]_10_ was incorporated into the PET‐19b3 expression vector in *Escherichia coli* BL21(DE3). The recombinant strains were grown at 37 °C in 500 mL flasks containing 100 mL of Luria‐Bertani medium for overnight culture in a shaking incubator at 250 rpm. A 100 mL seeding culture was transferred into 1L LB medium and 37 °C. Cells were induced with 1 mM IPTG when the optical density at 600 nm reached 0.6–0.8. After 6 h incubation, cells were harvested by centrifugation at 8500 rpm for 15 min at 4 °C. SELP was purified using the reverse temperature cycling method. The bacterial pellet was resuspended in PBS with lysozyme, and the cells were disrupted by sonication on ice. The cell lysate was cleared by centrifugation at 8000 rpm for 15 min at 4 °C, and then the supernatant containing SELP was diluted by 2X TN buffer, incubated at 70 °C for 2 h, and centrifuged at 5000 rpm for 5 min at 40 °C. Then, the supernatant was discarded, and the pellet containing SELP was recovered by deionized water at 4 °C followed by another cold spin at 8000 rpm for 15 min at 4 °C. The supernatant containing the purified SELPs was dialyzed (MWCO 3.5 kDa) against deionized water for 2 days. After lyophilization, the protein was stored at 4 °C for future use. The purity of the SELP proteins was determined via SDS‐PAGE.

### Preparation of Enzymatically Crosslinked SELP Hydrogels

SELP hydrogels were fabricated according to previous publications.^[^ ^27^ ^]^ The lyophilized SELP powder was dissolved in deionized water at 4 °C to prepare SELP stock solution. HRP powder was mixed with deionized water to form a stock solution with a concentration of 40 mg·mL^−1^. To prepare a 5% Silk hydrogel, 3 µL of HRP stock solution was mixed with t 100 µL Silk stock solution, and then the crosslinking reaction was triggered by adding 6 µL 1 wt.% H_2_O_2_ solution to the SELP/HRP mixture. After incubating at 4 °C overnight, enzymatically crosslinked SELP hydrogels were obtained.

### Preparation of Enzymatically Crosslinked Silk Hydrogels

50 g raw silk (Bombyx mori) was boiled in 0.05 m sodium carbonate solution for at least 30 min to remove the sericin layer. After a thorough cleaning and drying at ambient environment, degummed silk was obtained. 10 g of degummed silk was added into 50 mL of 9.3 m lithium bromide (LiCl) solution. The mixture was stirred at the temperature of 60 °C for 6 h, followed by dialysis against distilled water for at least 4 days to completely remove LiCl. Finally, the silk solution was frozen and lyophilized at −80 °C to acquire the silk foam. The lyophilized silk foam was dissolved in deionized water at 4 °C to prepare silk stock solution. To prepare a 5% Silk hydrogel, 3 µL of HRP solution was mixed with 100 µL silk stock solution, and then the crosslinking reaction was triggered by adding 3 µL 1 wt.% H_2_O_2_ solution to the Silk/HRP mixture. After incubating at 4 °C overnight, enzymatically crosslinked silk hydrogels were acquired.

### Preparation of Fe‐SELP

The Fe‐SELP was fabricated using a two‐electrode configuration with Fe electrode as the anode and Al plate as the cathode. Briefly, SELP hydrogel was soaked into 100 mm NaCl solution for at least 30 minutes. Then, superficial dry SELP hydrogel was sandwiched by Fe and Al electrodes, and a desired voltage was applied to the anode to generate and release ions into the SELP hydrogel. After electrochemical treatment, obtained gels were directly utilized for preparing Fe_3_O_4_‐SELP or lyophilized for further characterization.

### Preparation of Fe_3_O_4_‐SELP

The Fe_3_O_4_‐SELP was prepared using a modified in situ precipitation method.^[^ ^71^ ^]^ To acquire a bilayer structure, as‐prepared Fe‐SELP hydrogels were partially immersed into the phenylmethylsiloxane to expose the electrochemically treated surface. Then, the 1 m FeCl_3_·6H_2_O solution was carefully dropped to the surface of Fe‐SELP for 20 min. The content of Fe^3+^ was designed to meet the stoichiometric ratios of [Fe^3+^]: [Fe^2+^] = 2: 1. Next, the filter papers were immersed in the 6 m NaOH for at least 5 min. After draining the FeCl_3_·6H_2_O solution, NaOH‐immersed filter paper was immediately placed on the surface of the Fe‐SELP hydrogel for 30 minutes. This step was repeated twice. The whole reaction was processed in a Petri dish with a narrow space. A small amount of water was sprayed within the Petri dish to maintain a stable humidity environment. The final products were placed into the deionized water overnight to remove residue reagents. It was worth noting that the Fe_3_O_4_‐SELP robot could be tailored into different shapes by customizing the SELP hydrogel with different‐shaped PDMS molds.

### Preparation of Fe_3_O_4_‐SELP‐ExSitu

Fabricated SELP hydrogels were immersed into the solution of FeCl_3_·6H_2_O and FeCl_3_·4H_2_O with the molar ratio of [Fe^3+^]: [Fe^2+^] = 2: 1 for 20 min. After cleaning the mixed solution, the 6 m NaOH‐treated filter paper was placed on the surface of the Fe‐SELP hydrogel for 30 min. This step was repeated twice. The final products were placed into the deionized water overnight to remove residue reagents.

### Apparatus for Characterization

XRD equipped with a Cu Kα sealed tube was carried out to characterize the phase composition (D8 Advance powder diffractometer, Bruker, Germany). SEM (Gemini Sigma 300 VP, Zeiss, Germany) was employed to observe the sample morphologies. Fourier‐transform infrared (FT‐IR) spectroscopy was performed using an INVENIO‐S spectrophotometer (Bruker, Germany). A TEM (JEM‐1230, JEOL, Japan) operating at 120 kV was utilized to take higher magnification images of the iron oxide particles. XPS (K‐Alpha X, Thermo Fisher Scientific, USA) with monochromatic Al Kα radiation was performed to analyze the surface chemical states. ICP‐OES was conducted using a Thermo iCAP 7200 ICP‐OES instrument (Thermo Fisher Scientific, USA). VSM (LakeShore 7404, KES, USA) was employed to examine the magnetic properties of different samples. The UV‐vis‐NIR absorption spectra were tested using UV‐3600i Plus spectrophotometer (Shimadzu, Japan). The rheological measurements (Discovery HR20 rheometer, TA Instruments, USA) were performed to characterize the mechanical prosperities of different samples.

### Compression Tests

The mechanical properties were assessed using universal testing machines (ETM501, Wance, China) with a 50 N load cell. The stress–strain curves were measured in the compression mode at a crosshead speed of 0.1 mm min^−1^ in 4 °C. Young's modulus was calculated as the slope of the stress‐strain curves in the linear elastic region. At least three repeated experiments were conducted for each sample to reduce the test errors.

### In Vitro Degradation Test

SELP and Fe_3_O_4_‐SELP samples were incubated in 125 µg mL^−1^ trypsin at 37 °C for degradation studies. After rinsing with deionized water and drying, the remaining mass of the sample after incubation was recorded for degradation analysis.

### Detection of the Fe Content in the Fe_3_O_4_‐SELP

The Fe content was measured by ICP‐OES. Fe_3_O_4_‐SELP samples were incubated in aqua regia overnight. After complete dissolution, samples were centrifuged at 6000 rpm for 10 min. The liquid supernatants were used for the ICP‐OES test to evaluate the iron content.

### HUVECs Culture, Cell Proliferation, and Live/Dead Staining In Vitro

The HUVECs were cultured in low glucose DMEM supplemented with 10% fetal bovine serum and 1% penicillin/streptomycin. The cells were maintained in humidified air containing 5% CO_2_ at 37 °C, and the culture medium was changed every 2 d. The extract medium was prepared by immersing the samples into low glucose DMEM with the solid mass to liquid volume ratio of 0.2 g mL^−1^. After 24 h of leaching, the supernatant liquids were collected and diluted to 10% by supplementing fresh complete culture medium. To test the cell proliferation ability, the cells were seeded in 96‐well culture plates at a density of 4 × 10^3^ cells per well. After cell attachment, the complete culture medium was removed, and the cells were incubated with culture medium containing 10% extract medium. The cells were cultured for several days and detected using the Cell Counting Kit‐8 (CCK‐8, Cat No.40203; Yeasen, Shanghai, China). Similarly, for live and dead cell staining, the cells were seeded in 48‐well plates at a density of 1 × 10^4^ cells per well and cultured with DMEM medium containing 10% extract medium for 1 d. After this time, the cells were washed and treated with a serum‐free medium (200 µL) containing 1 µm calcein AM (APExBIO) and 4 µm PI. After incubation for 30 min at 37 °C, the cells were washed three times with PBS and observed under a fluorescence microscope (Olympus).

### Animal Experiments

Male SD rats (250–300 g) were used in this research, as approved by the Animal Ethics Committee of Zhejiang University (Serial number: ZJU20220533), and followed the guidance principles of the care and use of laboratory animals. The rats were anesthetized with isoflurane. After anesthesia, two 1 cm incisions were created on the rats’ dorsal skin, and small subcutaneous pockets were fabricated. The SELP hydrogels and Fe_3_O_4_‐SELP hydrogels were sterilized and implanted into the pockets. Incisions were closed with sutures. After 4 weeks, the rats were sacrificed, and the tissues in contact with the hydrogels were harvested for further tests.

### Histological Analysis and Assessment

The tissues were fixed in excessive 4% (w/v) paraformaldehyde overnight. Then, the tissues were dehydrated and embedded. Next, the implanted hydrogels with the surrounding tissues were sectioned with a thickness of 7 µm, and H&E staining was performed based on the standard protocols. Pannoramic MIDI scanner (3D HISTECH, Hungary) was employed to collect histological images.

### Comsol Simulation

Magnetic fields around a column magnet and the magnetic force field of the soft robot under magnetic fields were calculated using the commercial software COMSOL Multiphysics 6.2. Residual magnetization B_r_ = 800 mT is used as the input parameter.

### Fluorescence Excitation‐Emission Spectra

The fluorescence excitation‐emission spectra were collected using a Hitachi F4500 Spectrofluorometer (Hitachi, Schaumburg, IL). To test the excitation‐emission spectra of SELP hydrogel, 5% SELP precursor solution mixed with HRP and H_2_O_2_ was injected and gelled into the fluorescence cuvette. The spectra were processed to subtract the background fluorescence from the solvent and cuvette.

### Scanning Electron Microscopy

The microstructure of the hydrogels was assessed by SEM (Gemini Sigma 300 VP, Zeiss, Germany). Before the observation, hydrogels exposed to different equilibrium conditions were rapidly frozen with liquid nitrogen for 10 min to minimize the formation of crystalline ice. Afterward, the samples were freeze‐dried in a lyophilizer (SCIENTZ‐12N/C, Scientz Biotechnology, China). The dried hydrogels were subjected to cryofracture in liquid nitrogen to expose their cross‐sectional surfaces. Then, the cross‐sectional surface was sputtering coated using a gold target. Images were captured using SE2 detectors at 5 kV.

### Fourier Transform Infrared Spectroscopy

The hydrogel samples were rapidly frozen with liquid nitrogen and lyophilized for ATR‐FTIR measurements. For each measurement, 32 scans were co‐added with a resolution of 4 cm^−1^, and the wavenumbers ranged from 400 to 4000 cm^−1^. The background spectra were taken under the same conditions and subtracted from the sample scans.

### Differential Scanning Calorimetry

The thermodynamic properties were characterized by DSC (nano DSC, TA Instruments, USA). The instrument was equilibrated for 100 min, and the SELP samples were equilibrated at 4 °C before DSC measurements. To determine the LCSTs of SELP, the samples were equilibrated at the initial ramp temperature for 10 min. Following that, the sample was heated from 0 to 100 °C and cooled back to 0 °C in the sample chamber at a constant rate of 1 °C min^−1^. The baseline scans were collected using the same solvent under the same conditions and subtracted from the sample scans.

### Detection of the Fe Content in the Fe_3_O_4_‐SELP

The Fe content was measured by ICP‐OES. Fe_3_O_4_‐SELP samples were incubated in aqua regia overnight. After complete dissolution, samples were centrifuged at 6000 rpm for 10 min. The liquid supernatants were used for the ICP‐OES test to evaluate the iron content.

### Statistical Analysis

Statistical analysis consisted of a one‐way ANOVA carried out in GraphPad Instant software (GraphPad Software), followed by Duncan's multiple range test. Data are reported as mean ± standard deviation and statistical significance was accepted at *p* < 0.05.

## Conflict of Interest

The authors declare no conflict of interest.

## Author Contributions

W.H. and H.Z. conceived the idea and designed the experiments; H.Z. led the physical experiments and computational simulations; B.Y., D.Y., and T.J. contributed to material synthesis and characterization; K.N. contributed to animal experiments; J.T., J.O., and X.Y. contributed to data visualization; W.H. and Q.Z. supervised the study. All authors contributed to the figures and wrote the manuscript.

## Supporting information



Supporting Information

Supplemental Movie 1

Supplemental Movie 2

Supplemental Movie 3

Supplemental Movie 4

Supplemental Movie 5

Supplemental Movie 6

Supplemental Movie 7

## Data Availability

The data that support the findings of this study are available from the corresponding author upon reasonable request.
