# Supplementary material for: Electrochemical‐Genetic Programming of Protein‐Based Magnetic Soft Robots for Active Drug Delivery
Source: Adv Sci (Weinh). 2025 Apr 29;12(27):2503404. doi: 10.1002/advs.202503404 (PMC12279187; doi:10.1002/advs.202503404)
Supplement: Supplementary file 1 — Supporting Information [file ADVS-12-2503404-s008.pdf]

ADVANCED SCIENCE
Open Access

Supporting Information

for *Adv. Sci.*, DOI 10.1002/advs.202503404

Electrochemical-Genetic Programming of Protein-Based Magnetic Soft Robots for Active Drug Delivery

*Hang Zhao, Bo Yu, Dingyi Yu, Ting Ji, Kexin Nie, Jingyi Tian, Xinchen Shen, Kaiyue Zhang, Junhan Ou, Xinyi Yang, Dongfang Xiao, Qi Zhou and Wenwen Huang**

Supporting Information

Title

Electrochemical-genetic programming of protein-based magnetic soft robots for active drug delivery

*Hang Zhao[1], Bo Yu[1], Dingyi Yu[1,2], Ting Ji[1], Kexin Nie[1,2], Jingyi Tian[1], Xinchen Shen[1,2], Kaiyue Zhang[1], Junhan Ou[1], Xinyi Yang[1], Dongfang Xiao[1], Qi Zhou[2], Wenwen Huang[1,2,3,4,5,6]**

[1]Centre for Regeneration and Cell Therapy, The Zhejiang University-University of Edinburgh Institute, Zhejiang University School of Medicine, Zhejiang University, Hangzhou 310058, China

[2]Deanery of Biomedical Sciences, Edinburgh Medical School, College of Medicine and Veterinary Medicine, The University of Edinburgh, Edinburgh EH89AG, UK

[3]Department of Orthopedics of the Second Affiliated Hospital, Zhejiang University School of Medicine, Zhejiang University, Hangzhou 310058, China

[4]Dr. Li Dak Sum & Yip Yio Chin Center for Stem Cells and Regenerative Medicine, Zhejiang University School of Medicine, Zhejiang University, Hangzhou 310058, China

[5]State Key Laboratory of Biobased Transportation Fuel Technology, Zhejiang University, Hangzhou 310027, China

[6]Biomedical and Health Translational Research Centre of Zhejiang Province, Zhejiang University, Hangzhou 310003, China

*Corresponding author.

E-mail: wenwenhuang@intl.zju.edu.cn

Table of Contents

Photothermal conversion efficiency calculation

Magnetic actuation of Fe_3O_4-SELP robots

Supplementary Figures 1 to 27

Supplementary Movies 1 to 7

Supplementary Tables 1

Supplementary References

Photothermal conversion efficiency calculation

The Fe_3O_4-SELP powders were dispersed in the distilled water and prepared as a solution with a concentration of 1 mg/ml. As-prepared solution was irradiated by 1.2 W cm^{-2} NIR laser for 5 min to calculate the photothermal conversion efficiency. The following equation determined the photothermal conversion efficiency (η):

$$\eta = \frac{hS(T_{max} - T_{surr}) - Q_{dis}}{I(1 - 10^{-A_{808}})} \qquad \text{equation 1}$$

where h, S, T_{max}, T_{surr}, Q_{dis}, I, A_{808} are heat transfer coefficient, irradiated area, the maximum system temperature, the environmental temperature, heat dissipation from the environment, laser power density and absorbance of the sample at 808 nm, respectively.

Specifically, the value of *hS* was obtained from the equation below:

$$\tau_s = \frac{mC_p}{hS} \qquad \text{equation 2}$$

where τ_s, m, and C_p are the time constant, mass of the system, and heat capacity (4.2 J g^{-1} K^{-1}).

According to the equations below, τ_s was determined by analyzing the cooling curves.

$$t = -\tau s(ln\theta) \qquad \text{equation 3}$$

$$\theta = \frac{T - Tsurr}{Tmax - Tsurr} \qquad \text{equation 4}$$

T: the temperature corresponding to time (t) in the cooling curve after the light source was turned off.

Furthermore, Q_{dis} was calculated from the formula below:

$$Q_{dis} = hS\left(T_{max,water} - T_{surr,water}\right) \qquad \text{equation 5}$$

Magnetic actuation of Fe_3O_4-SELP robots

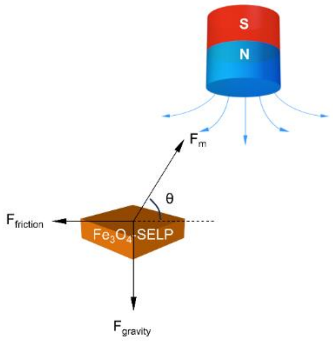

Schematic illustration of the forces exerted on Fe_3O_4-SELP.

The motion of Fe_3O_4-SELP robot relies on magnetic actuation of a permanent magnet, which generates an external magnetic field $\boldsymbol{H}$ to impose a force and a torque on the robot: $F_m = \mu_0 V(M \cdot \nabla)H, T_m = \mu_0 VM \times H$ equation 6

where $\mu_0 = 4\pi \times 10^{-7}$ T·m·A^{-1} is the permeability of free space and $\boldsymbol{M}$ is the magnetization of the Fe_3O_4-SELP robot of body volume V. As our Fe_3O_4-SELP robot is determined to be paramagnetic, its $\boldsymbol{M}$ aligns with $\boldsymbol{H}$ when actuated and the magnitude can be calculated as: $M = \rho M_S$, where M_S is the measured saturation magnetization and ρ is density of Fe_3O_4-SELP.

To enable translational motion, the pulling force tangential to the surface needs to overcome the fiction force: $F_m COS\theta - F_{friction} \geq 0$ equation 7

where $F_{friction} = \mu(mg - F_m sin\theta)$. equation 8

for given friction coefficient of the surface μ under gravity$g$.

Combing all above, we can arrive at the criterion for effective magnetic actuation:

$M_S \nabla H \geq \mu g/\mu_0(\mu sin\theta + cos\theta)$ equation 9

Based on this criterion, the robot's locomotion capability can be enhanced either through increasing the saturation magnetization of Fe_3O_4-SELP (e.g., with higher Fe_3O_4 density during fabrication) or the magnetic field gradient of the column magnet (with a stronger permanent magnet for control).

Supplementary Figures

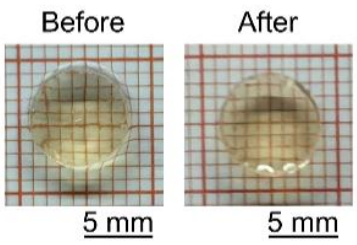

Figure S1. Digital photograph of S_4E_8Y hydrogels before (4 ºC) and after (50 ºC) heat treatment for 15 min.

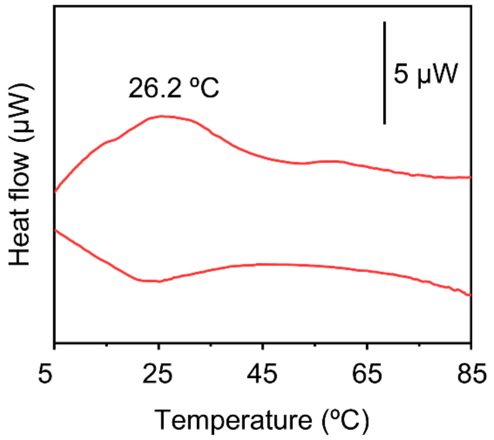

Figure S2. DSC heat flow versus temperature curves of SELP molecules.

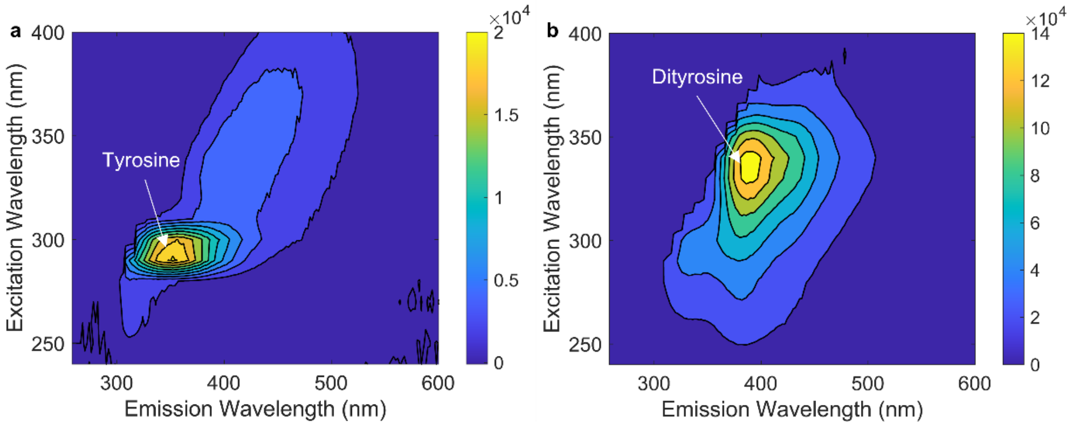

Figure S3. Fluorescence excitation-emission matrices of a) SELP solution and b) SELP hydrogel.

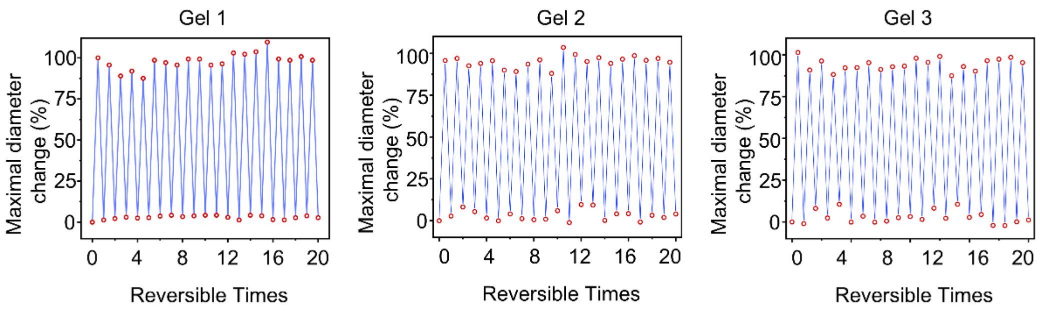

Figure S4. Cyclic stability of the SELP hydrogels under temperature change from 4ºC to 50ºC at aqueous environment.

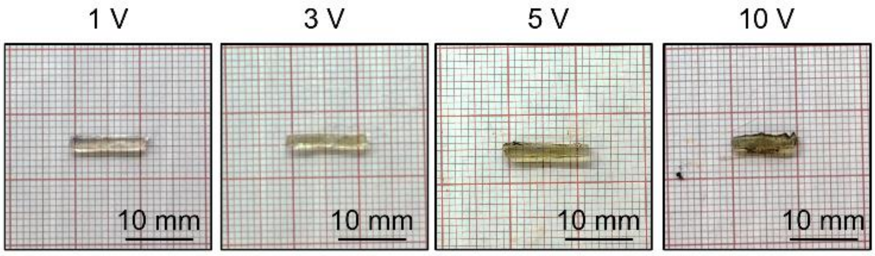

Figure S5. Optical images of the SELP hydrogel after different voltage treatments.

SELP hydrogels were treated with different voltages under the condition of constant processing time. There was negligible difference in SELP hydrogels after 1V treatment. As the processing voltage increased, the color of SELP surface was changed significantly. When the processing voltage reached 10V, the hydrogel structure collapsed due to the violent electrode reactions.

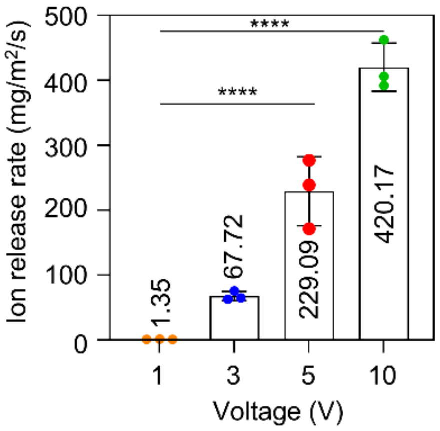

Figure S6. The ion release rate under different voltage treatments.

The Fe^{2+} release rate was quantified from the equation below:

$$\frac{M_{Fe}}{St} \qquad \text{equation 10}$$

Where M_{Fe}, S, t are the content of Fe^{2+} ion release, the contact area between electrode and hydrogel, and the electrode processing time, respectively.

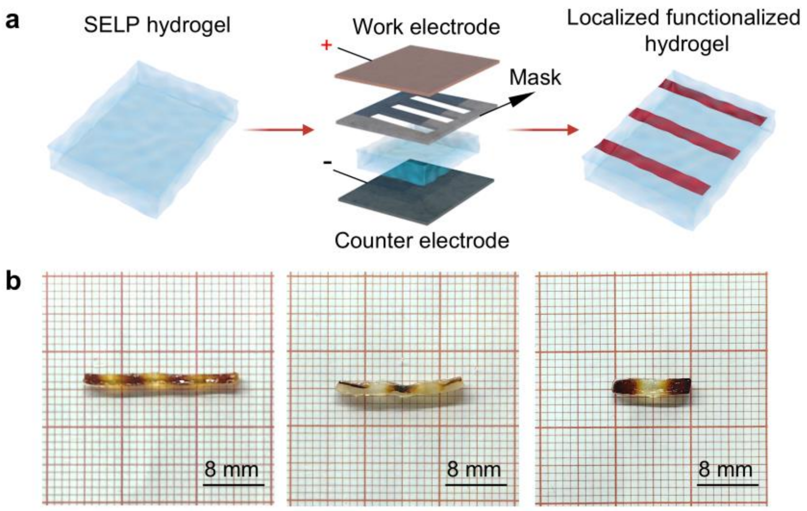

Figure S7. a) Schematic illustration of the fabrication strategy of localized patterning magnetic species on the SELP hydrogel surface. b) Optical images of patterned Fe_3O_4-SELP hydrogel.

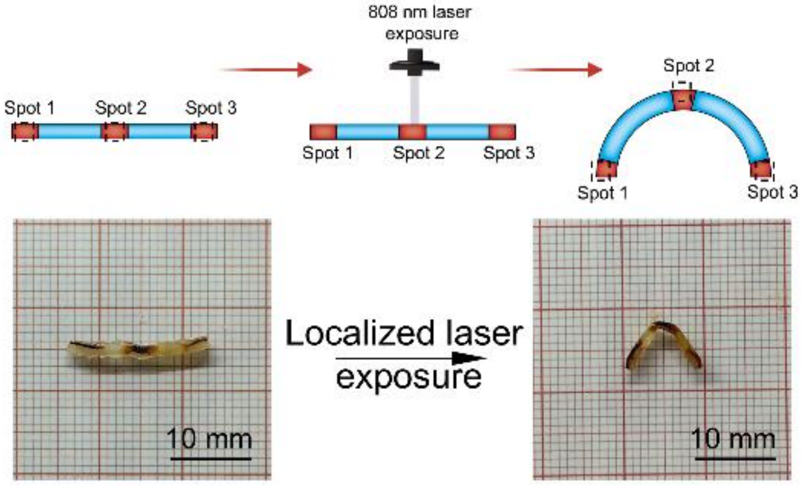

Figure S8. Representative photographs of patterned Fe_3O_4-SELP hydrogel before and after localized photothermal treatment using 808 nm laser irradiation.

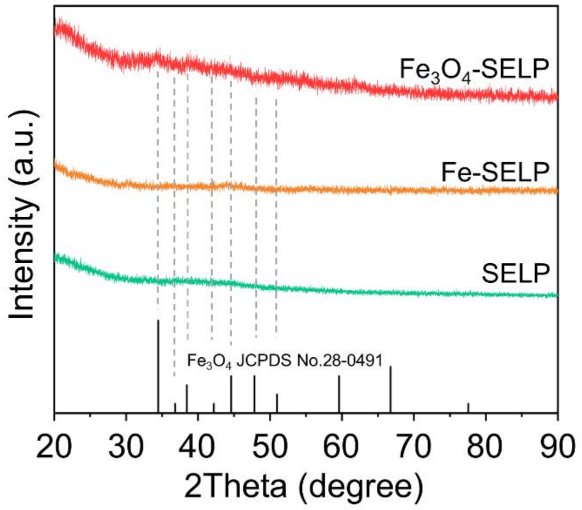

Figure S9. XRD patterns of the SELP, Fe-SELP and Fe_3O_4-SELP.

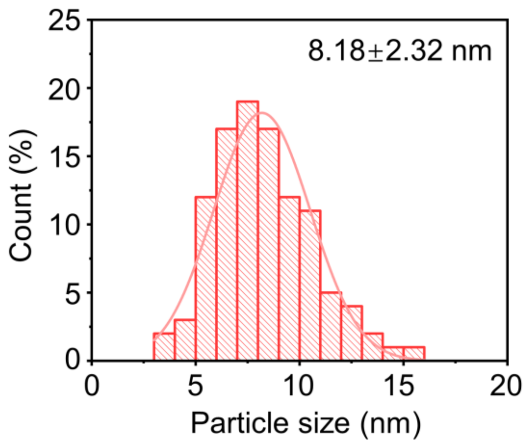

Figure S10. Size distribution of synthesized Fe_3O_4 nanoparticles.

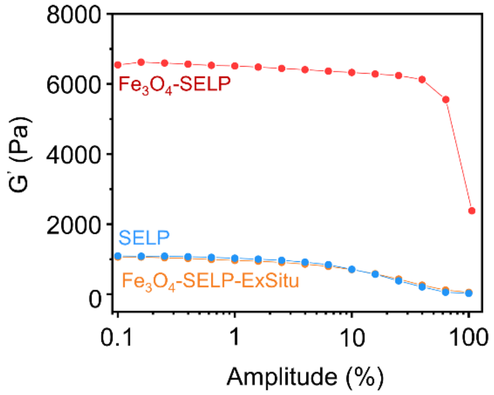

Figure S11. The representative strain amplitude sweeps of SELP, Fe_3O_4-SELP and Fe_3O_4-SELP-ExSitu hydrogels.

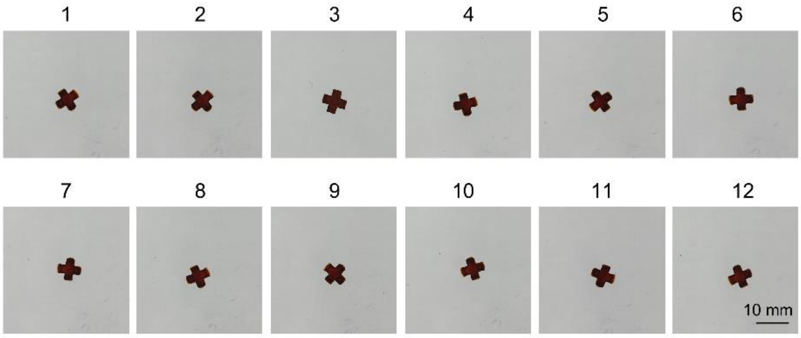

Figure S12. Optical images of Fe_3O_4-SELP robots after release from catheter. The number above images indicates the repetition times of catheter releasing process.

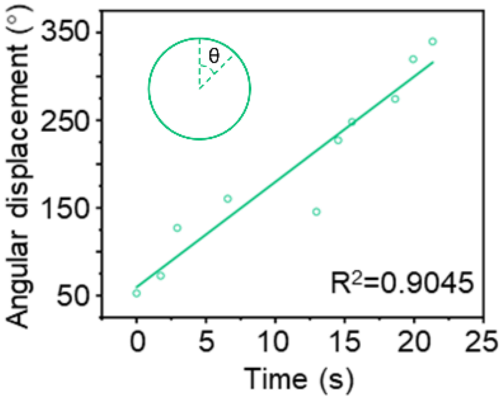

Figure S13. The angular displacement curve of Fe_3O_4-SELP robots as a function of time and simulated using a linear fitting. The insertion shows the definition of angular displacement.

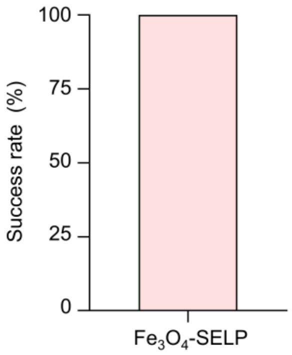

Figure S14. The success rate of the Fe_3O_4-SELP robot movement in maze navigation.

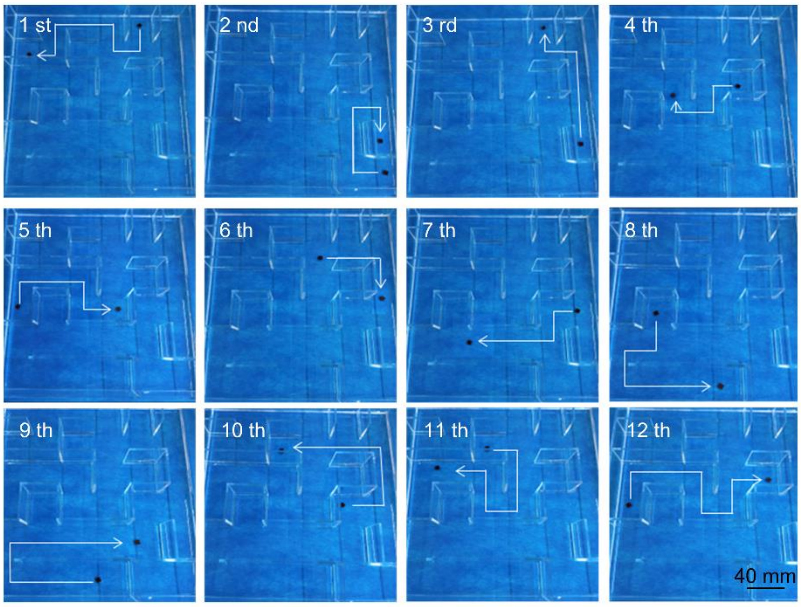

Figure S15. The trajectory of the Fe_3O_4-SELP robot movement in maze navigation.

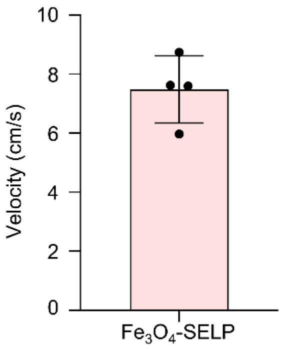

Figure S16. The velocity of the Fe_3O_4-SELP robot movement in maze navigation.

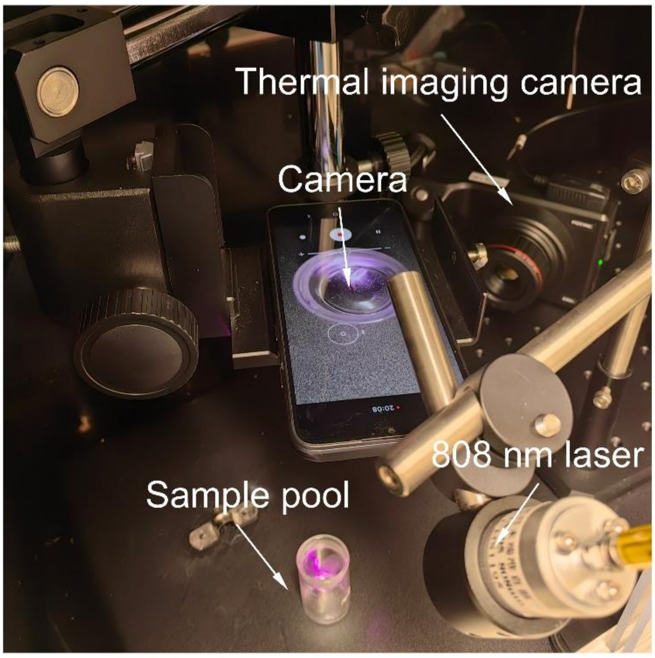

Figure S17. Lab-designed apparatus used to *in situ* observe NIR triggered drug release performance of the magnetic Fe_3O_4-SELP robot. The setup is equipped with a camera, infrared thermography, an 808 nm laser, and a sample pool.

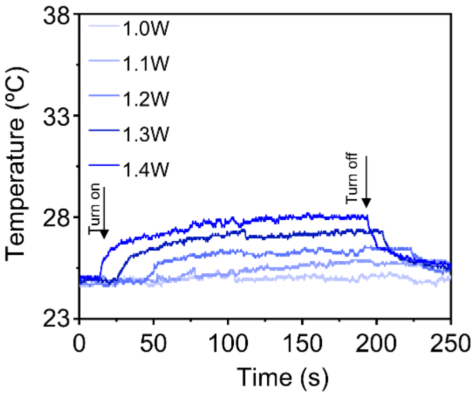

Figure S18. Photothermal conversion of SELP under 808 nm laser irradiation with different exposure intensities (1.0-1.4 W cm^{-2}).

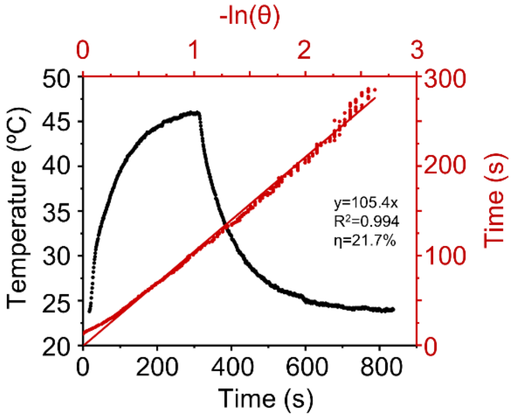

Figure S19. Heating-cooling cycle and linear fitting of time versus – ln(θ) obtained from the cooling curve of Fe_3O_4-SELP.

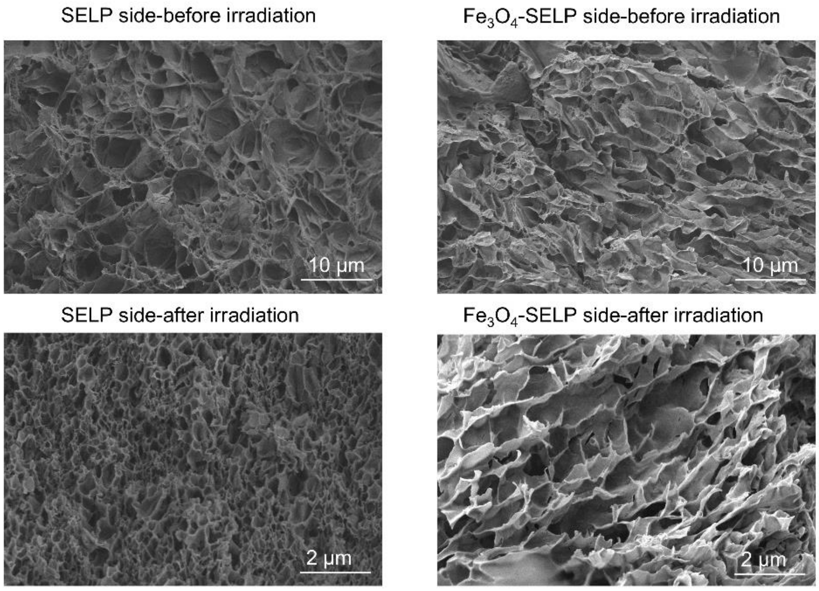

Figure S20. SEM images of the SELP and Fe_3O_4-SELP before and after irradiation.

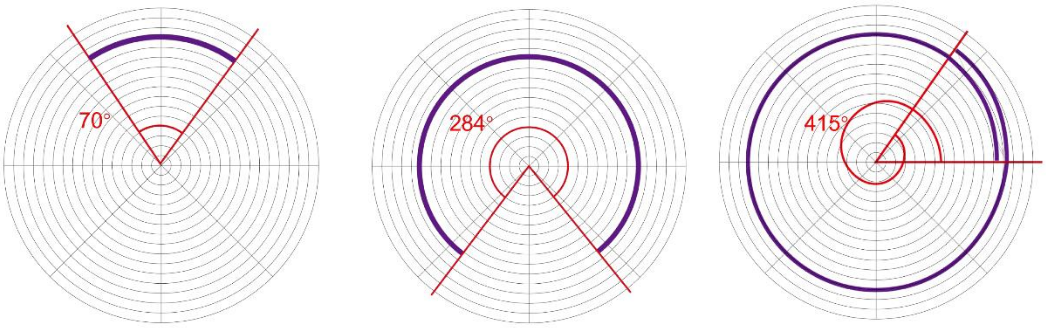

Figure S21. The definition of bending angle.

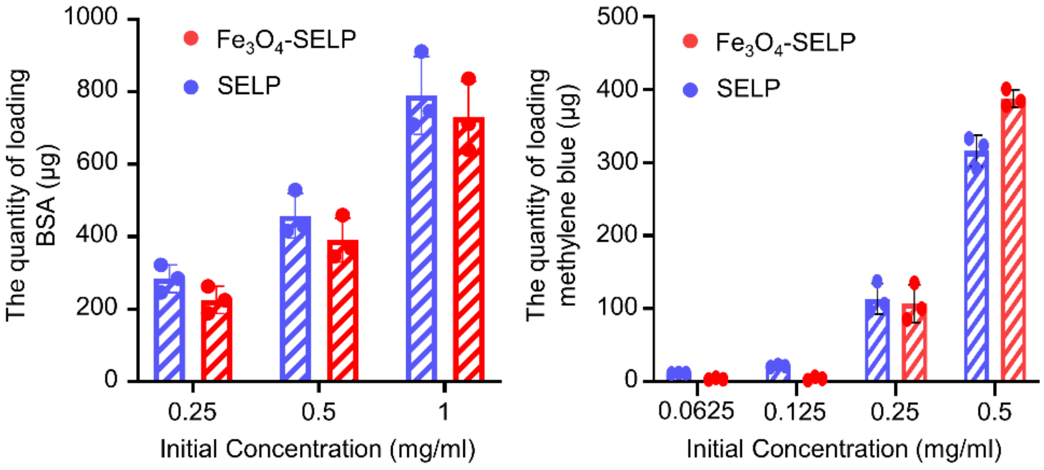

Figure S22. BSA and Methylene blue loading of SELP and Fe_3O_4-SELP robots.

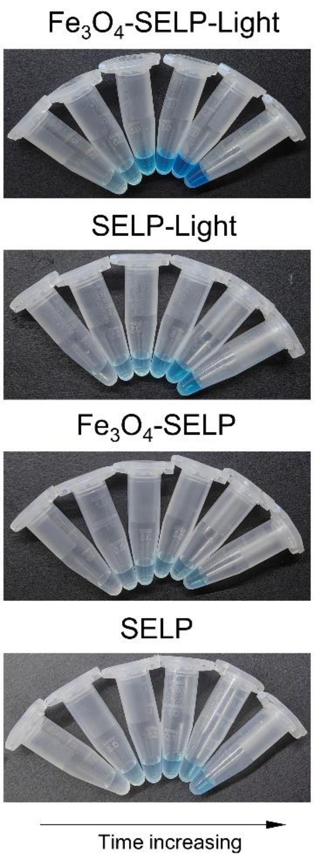

Figure S23. Time-dependent Methylene blue release of SELP and Fe_3O_4-SELP robots with or without NIR irradiation.

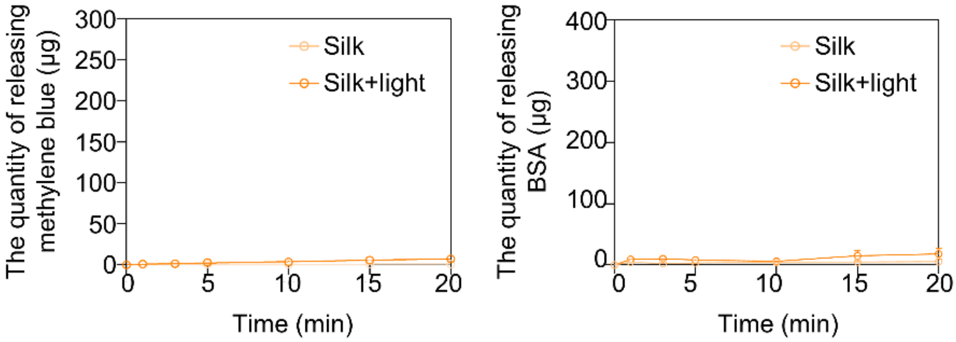

Figure S24. Methylene blue and BSA release profile of silk hydrogel with or without 808 nm laser irradiation.

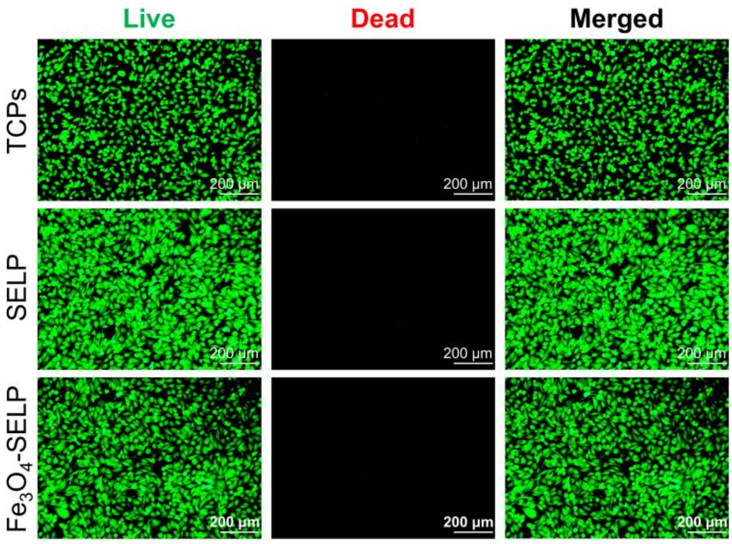

Figure S25. Live/dead cellular staining images of HUVECs incubated with the extract media from the tissue culture plate (TCP), SELP, and Fe_3O_4-SELP robots for 24 h, respectively. Live cells were stained green and dead cells were stained red.

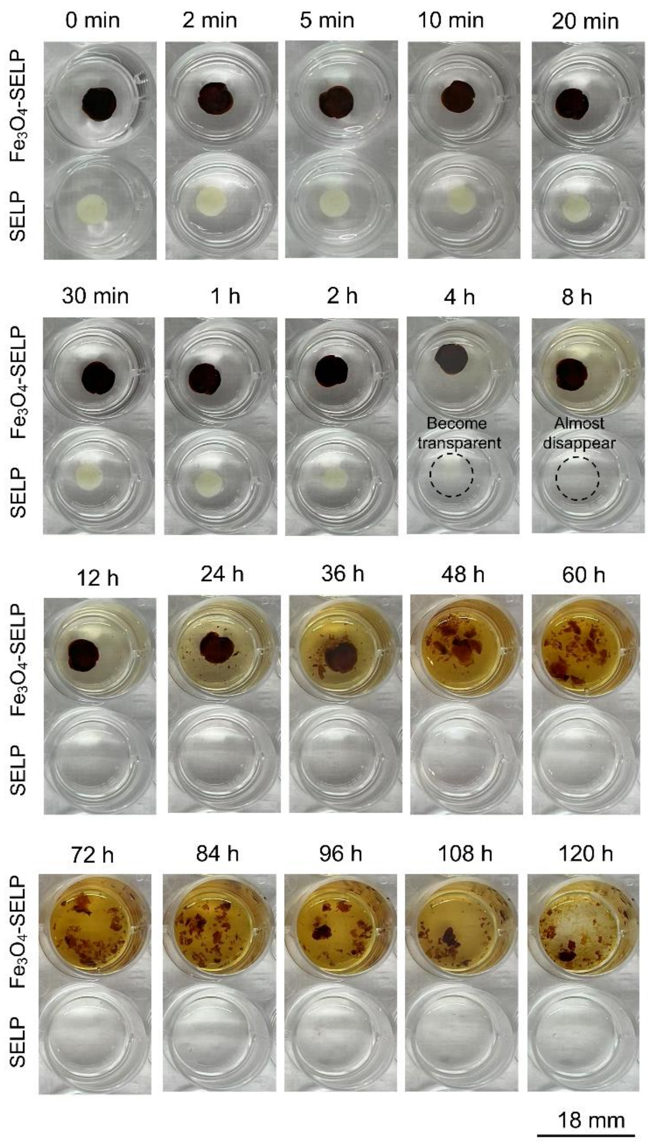

Figure S26. Degradation of SELP and Fe_3O_4-SELP robots with trypsin treatment.

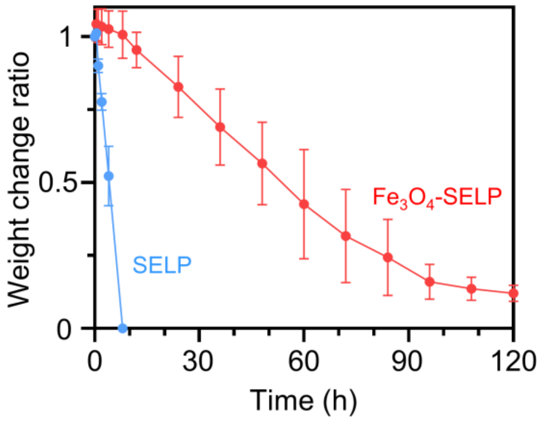

Figure S27. The weight change ratio of SELP and Fe_3O_4-SELP robots during the degradation process in trypsin solution.

Supplementary Movies

Movie S1. The release behavior of Fe_3O_4-SELP robots from a capsule.

Movie S2. The catheter encapsulation of Fe_3O_4-SELP robots.

Movie S3. The release behavior of Fe_3O_4-SELP robots from the catheter.

Movie S4. The rolling behavior of Fe_3O_4-SELP robots.

Movie S5. The flipping behavior of Fe_3O_4-SELP robots.

Movie S6. Maze navigation of Fe_3O_4-SELP robots.

Movie S7. Targeted navigation and drug release performance of the Fe_3O_4-SELP robot in *ex vivo* intestinal tract.

Supplementary tables

Table S1. Mechanical properties of PNIPAM-based system and Fe_3O_4-SELP

**Materials**	**Mechanical properties**	**Reference**
SELP+ Fe_3O_4	73.91kPa, G'=6 kPa	Our work
PNIPAM	3.96 kPa	[1]
P(NIPAM-co-AM)+PVA+Fe_3O_4	56 kPa	[2]
PNIPAM+BIS+ Fe_3O_4	30 kPa	[3]
PNIPAM-Ag-PEDOT	1.63 kPa	[4]
PNIPAM+silica-coated carbonyl iron particles	G'=3.6 kPa	[5]
PNIPAM+PEI	G'=850 Pa	[6]
PNIPAM+MBAAm	13.9 kPa	[7]
PNIPAM+clay	9.9 kPa	[8]
PNIPAM+heparin+starPEG	60 kPa	[9]
PNIPAM	34.4 kPa	[10]

Supplementary References

[1] X.-Z. Zhang, D.-Q. Wu, C.-C. Chu, Biomaterials 2004, 25, 3793.

[2] H. Zhou, Z. Jin, Y. Gao, P. Wu, J. Lai, S. Li, X. Jin, H. Liu, W. Chen, Y. Wu, A. Ma, Colloids and Surfaces A: Physicochemical and Engineering Aspects 2022, 636, 128113.

[3] L. Xu, S. C. Lamont, T. Li, Y. Zhang, W. Pan, C. Gao, C. Zhu, S. Chen, H. Hu, J. Ding, F. J. Vernerey, ACS Macro Lett. 2023, 12, 549.

[4] D. Naranjo, S. Paulo-Mirasol, S. Lanzalaco, E. Armelin, J. García-Torres, J. Torras, Advanced Sustainable Systems 2024, 8, 2400234.

[5] L. Selzer, S. Odenbach, Journal of Magnetism and Magnetic Materials 2020, 501, 166394.

[6] C. Ma, Y. Shi, D. A. Pena, L. Peng, G. Yu, Angewandte Chemie International Edition 2015, 54, 7376.

[7] T. R. Matzelle, G. Geuskens, N. Kruse, Macromolecules 2003, 36, 2926.

[8] K. Haraguchi, T. Takehisa, Advanced Materials 2002, 14, 1120.

[9] J. Sievers, S. Zschoche, R. Dockhorn, J. Friedrichs, C. Werner, U. Freudenberg, ACS Appl. Mater. Interfaces 2019, 11, 41862.

[10] C. Norioka, A. Kawamura, T. Miyata, Polym. Chem. 2017, 8, 6050.
